# Nanoemulsions Based on Soluble Chenopodin/Alginate Complex for Colonic Delivery of Quercetin

**DOI:** 10.3390/antiox13060658

**Published:** 2024-05-27

**Authors:** Arturo Intiquilla, Migdalia Arazo, Alexander Gamboa, Nelson Caro, Martin Gotteland, Alan Palomino-Calderón, Lilian Abugoch, Cristian Tapia

**Affiliations:** 1 Laboratorio de Biología Molecular, Facultad de Farmacia y Bioquímica, Universidad Nacional Mayor de San Marcos, Jirón Puno 1002, Lima 15081, Peru; aintiquillaq@unmsm.edu.pe; 2 Departamento de Ciencia de los Alimentos y Tecnología Química, Facultad de Ciencias Químicas y Farmacéuticas, Universidad de Chile, Santos Dumont 964, Santiago 8330015, Chile; alan.palomino@ug.uchile.cl; 3 Departamento de Ingeniería Química y Bioprocesos, Facultad de Ingeniería, Pontificia Universidad Católica de Chile, Avda. Vicuña Mackenna 4860, Macul, Santiago 8330015, Chile; migdi.arazo@ucl.cl; 4 Facultad de Química y Biología, Universidad de Santiago de Chile, Av. Libertador Bernardo O’Higgins 3363, Estación Central, Santiago 9170022, Chile; alexander.gamboa@usach.cl; 5 Centro de Investigación Austral Biotech, Facultad de Ciencias, Universidad Santo Tomás, Avenida Ejército 146, Santiago 8370003, Chile; nelsoncarofu@santotomas.cl; 6 Departamento de Nutrición, Facultad de Medicina, Universidad de Chile, Santiago 8330015, Chile; mgottela@uchile.cl; 7 Laboratorio de Microbiología y Probióticos, Instituto de Nutrición y Tecnología de Alimentos (INTA), Universidad de Chile, Santiago 8330015, Chile

**Keywords:** nanoemulsion, chenopodin/alginate, colon, quercetin

## Abstract

Inflammatory bowel disease (IBD) is an autoimmune disorder caused by uncontrolled immune activation and the subsequent destruction of the colon tissue. Quercetin (Qt) is a natural antioxidant and anti-inflammatory agent proposed as an alternative to mitigate IBD. However, its use is limited by its low oral bioavailability. This study aimed to develop nanoemulsions (NEs) based on a soluble chenopodin/alginate (QPA) complex and Tween 80 (T80), intended for the colonic release of Qt, activated by the pH (5.4) and bacteria present in the human colonic microbiota. NEs with different ratios of QPA/Tw80 (F1-F6) were prepared, where F4Qt (60/40) and F5Qt (70/30) showed sizes smaller than 260 nm, PDI < 0.27, and high encapsulation efficiency (>85%). The stability was evaluated under different conditions (time, temperature, pH, and NaCl). The DSC and FTIR analyses indicated hydrophobic and hydrogen bonding interactions between QPA and Qt. F4Qt and F5Qt showed the greater release of Qt in PBS1X and Krebs buffer at pH 5.4 (diseased condition), compared to the release at pH 7.4 (healthy condition) at 8 h of study. In the presence of *E. coli* and *B. thetaiotaomicron*, they triggered the more significant release of Qt (ƒ2 < 50) compared to the control (without bacteria). The NEs (without Qt) did not show cytotoxicity in HT-29 cells (cell viability > 80%) and increased the antioxidant capacity of encapsulated Qt. Therefore, these NEs are promising nanocarriers for the delivery of flavonoids to the colon to treat IBD.

## 1. Introduction

Inflammatory bowel disease (IBD) is a chronic inflammatory disorder of the gastrointestinal tract (GIT), encompassing ulcerative colitis (UC) and Crohn’s disease (CD) [1]. IBD has a complex etiology and pathophysiology, which limits its treatment, and is characterized by a microenvironment with high levels of reactive oxygen species (ROS), the elevated secretion of proinflammatory mediators (IL-1β, IL-6, TNF-α), a lower pH (2.3–5.5), and the overexpression of some enzymes (α-amylase, elastase, myeloperoxidase, etc.) [2,3,4]. Anti-inflammatories, both non-steroidal and steroidal, are frequently used for its treatment. However, they have serious side effects, such as hypertension, diabetes, a low bone mass, and osteoporosis [3,5]. Additionally, the colonic microbiota is altered in IBD, reducing the levels of the phyla Bacteroidetes (~67%) and Firmicutes (~28%) and increasing the levels of Proteobacteria (2%) and Actinobacteria (1%) [1].

Natural antioxidants and anti-inflammatory flavonoids (rutin, quercetin) do not produce side effects, and they are an effective alternative to attenuate IBD [3,6]. However, when administered orally, they trigger adverse conditions in the gastrointestinal tract (gastric fluid, digestive enzymes, and microbiota), which counteract their curative effects [7]. The encapsulation of active ingredients in nanoparticles (NPs) is an effective alternative that can improve the oral administration of these bioactive compounds, protecting them in the upper gastrointestinal tract and favoring their passive accumulation in inflamed tissue through the enhanced epithelial permeability and retention (eEPR) effect [8]. Furthermore, inflammation in the colonic tissue alters the integrity of the epithelial layer, decreasing the barrier function and increasing the permeability of NPs (10–1000 nm) [3].

Nanoemulsions (NEs) are kinetically stable nanocarriers (20 to 500 nm) that are derived from the mixture of two immiscible liquids (e.g., oil and water), stabilized by a surfactant [9]. NEs are a suitable alternative for the delivery of hydrophobic bioactives with therapeutic potential because they increase their solubility and stability, reducing or eliminating the need for toxic cosolvents (acetone, methanol, dimethyl sulfoxide) [10,11]. In previous studies, we developed oil/water (*o*/*w*) NEs using the nonionic synthetic surfactant Tween 80 (Tw80) in a volume of 10% (*w*/*w*) to encapsulate hydrophobic compounds such as thymol. Appropriate parameters in terms of size (<200 nm), polydispersity index (PDI) < 0.2, and encapsulation efficiency (67%) were obtained [12]. However, nowadays, there is great interest in the replacement of synthetic surfactants with ecological and sustainable natural molecules such as saponins, phospholipids, polysaccharides, proteins, or combinations of them, without compromising the quality of the product [13]. Among them, soluble protein/polysaccharide complexes are an attractive alternative because they can stabilize NEs through different physicochemical mechanisms (such as electrostatic and steric repulsion) [14]. In particular, Zhou et al. [9] developed NEs with a size < 300 nm, PDI < 0.27, and zeta potential of −33 mV, using a soy protein/alginate complex (SPI/SA) as a surfactant at concentrations in the range of 0.02–2.0% (*w*/*v*) at pH 7.0. The ATR–FTIR analysis confirmed the interaction of the SPI/SA complex with the oil phase via hydrogen bonds, evidenced by the broad band between 3000 and 3600 cm^−1^ and electrostatic interactions due to the decrease in and coupling of the carboxylate group of SA and the blue shift of the amide I (1655 cm^−1^) and amide II (1540 cm^−1^) bands of SPI, strengthening the SPI/SA complex surrounding the nanodroplet. In previous studies, we developed a protein/polysaccharide complex using chenopodin or quinoa 11S globulin (QP), extracted via isoelectric precipitation, with sodium alginate (Alg), in proportions of 1:4, 2:3, 1:1, 3:2, and 4:1 of QP-Alg (QPA). The intermolecular interactions between the biopolymers through hydrogen, hydrophobic, and electrostatic bonds were identified via ATR–FTIR, highlighting their potential as nanovehicles due to the hydrophilic and hydrophobic regions throughout the complex, mainly at the proportions of 4:1 and 3:2, based on fluorescence, surface hydrophobicity, and circular dichroism analyses [15]. Additionally, QPA complexes using QP isolated via the DF/UF crossflow filtration process showed similar behavior to QPA complexes using QP obtained via isoelectric precipitation [16,17].

NEs based on protein/polysaccharide complexes are an appropriate strategy for the controlled release of active ingredients in the colonic tissue due to their mucoadhesiveness, sensitivity to pH, and biodegradability (proteases, glucuronidases, and others) by the colonic microbiota [2]. This work aimed to prepare NEs composed of the QPA 3:2 complex and Tween 80 in proportions of 0/100, 20/80, 40/60, 60/40, 70/30, and 80/20 *w*/*w* of QPA/Tw80, for the controlled release of quercetin (Qt), used as a flavonoid model. The stability of the NEs was evaluated at different times (1–30 days), temperatures (4.0 and 25 °C), pH levels (2.0 to 8.0), and NaCl concentrations (100–500 mM), and the main interaction mechanisms between QPA and Qt were determined via ATR–FTIR. The performance of the NEs was evaluated against that of compendial PBS1X and biorelevant Krebs buffer at pH 7.4 (healthy condition) and 5.4 (disease condition) and in the presence of *Bacteroides thetaiotaomicron* and *Escherichia coli*. Finally, the cytotoxicity of the NEs and their antioxidant capacity were evaluated in the HT-29 cell line with oxidative stress induced by H_2_O_2_. In this study, QP was used for the first time in a chenopodin/alginate complex for the preparation of NEs intended for the release of drugs in the colon, activated by the pH and the bacteria of the human colon microbiota.

## 2. Materials and Methods

### 2.1. Materials

Quinoa flour (*Chenopodium quinoa* Willd.) was provided by Promauka Ltda-VI Region-Chile, with the following proximal composition (100 g): moisture 11.3 g, protein 11.2 g, carbohydrates 72.6 g, fat 4.9 g, and ash 55.5 mg (AOAC, 1995). The flour was stored at 4 °C until use. Medium-chain triglycerides (MCT) were obtained from Sasol Germany GmbH (Witten, Germany); Tween 80 and ethanol were purchased from Merck (Darmstadt, Germany). Low-viscosity alginic acid sodium salt from Macrocystis pyrifera (average molecular weight of 162 kDa and a 1.2 M/G ratio), ABTS, 6-hydroxy-2,5,7,8-tetramethylchroman-2-carboxylic acid (Trolox), disodium fluorescein (FL), 2,2′-azobis (2-amidinopropane dihydrochloride) (AAPH), and quercetin (≥95%, HPLC grade) were purchased from Sigma Aldrich (St. Louis, MO, USA).

The HT-29 Glc-/+ human colonic adenocarcinoma cell line (passages 51–54) was cultured in Dulbecco’s modified Eagle’s medium (DMEM), supplemented with 10% fetal bovine serum (SFB) and penicillin/streptomycin 1.0% (HyClone, Logan, UT, USA). Cells were subcultured with trypsin at 80–90% confluence (HyClone, Logan, UT, USA). The chemical reagents 3-(4,5-dimethylthiazol-2-yl)-2,5-diphenyltetrazolium (MTT), 2′,7′-dichlorofluorescein diacetate (DCFDA), and dimethylsulfoxide (DMSO) were purchased from Sigma Aldrich (St. Louis, MO, USA).

### 2.2. Isolation of Chenopodin

Chenopodin (QP) was obtained by following the methodology of Arazo et al. [16], with slight modifications. Defatted flour was suspended in 50 mM Tris–HCl buffer pH 8.3/0.5 M NaCl at a ratio of 1:10 (*w*/*v*), stirred for 1 h, and centrifuged at 13,000 rpm for 30 min (HermLe, model Z323K, Oststeinbek, Germany). The supernatant was filtered through a 0.45 µm membrane (Durapore, PVDF Millipore, Burlington, MA, USA) and concentrated in Chenopodin via DF/UF crossflow filtration (SARTOFLOW^®^ Slice 200 Benchtop Crossflow System Ultrafiltration, Goettingen, Germany), provided with 100 and 30 kDa cut-off membranes (Sartocon^®^ Slice 200 Hydrosart^®^ stabilized cellulose membrane cassettes, Bohemia, NY, USA) with a 0.02 m^2^ working area. The TMP was fixed at 9.72 kPa, and, for the diafiltration step, 8 volumes were used with both membranes, followed by a final concentration step using a 100–30 kDa cut-off membrane. The results obtained from the diafiltration and concentration processes were analyzed by electrophoresis according to Laemmli [18]. The fouling of the membrane during the protein concentration stage was studied according to the mathematical models of Hermia [19].

### 2.3. Preparation of Soluble Chenopodin/Alginate Complex (QPA)

The soluble QPA complex was prepared by following the methodology of Romo et al. [15]. Briefly, stock solutions of 2% (*w*/*v*) alginate (Milli-Q water) and chenopodin (adjusted to pH 8.0) were prepared. A mixture of the stock solutions was produced at a 3:2 ratio of chenopodin to alginate, finally adjusting the pH to 6.0 and stirring the mixture for 1.0 h.

### 2.4. Nanoemulsion Preparation

The NEs were prepared according to Robledo et al. [12], with modifications. The ratio of oil/water used was 1:9, and the ratio of surfactant/oil (SOR) was kept at 0.2. Under constant stirring, 2.5 g of the oily phase containing MCT (1.25 g), ethanol (1.225 g), and quercetin (0.025 g) was added to 22.5 g of the aqueous phase, containing a mixture of QPA/Tw80 (0.5 g) at different proportions of 0/100 (F1), 20/80 (F2), 40/60 (F3), 60/40 (F4), 70/30 (F5), and 80/20 (F6) *w*/*w*, at pH 6.0. The mixture was homogenized with an Ultra-Turrax T25 at 18,500 rpm for 3 min and transferred to a sonicator (Q-Sonica, Q700, Newtown, CT, USA) equipped with a probe with a 127 mm diameter, and it was sonicated at a frequency of 20 kHz and 700 W for 20 min, with a 25% amplitude and pulses (on time/off time) of 10 s on/15 s off in a cold bath to maintain a temperature of 25 °C. As a protein control in the NEs designated as P4 and P5, QPA was replaced with QP in the formulation.

### 2.5. Characterization of NEs

#### 2.5.1. Size, Polydispersity Index, and Zeta Potential

The particle hydrodynamic diameter, PDI, and zeta potential were measured at 25 °C using a Zetasizer Nano ZS-20 (Malvern Instruments, Malvern, UK) operating at 4.0 mW and 633 nm, with a fixed scattering angle of 173°. For the measurement, the NEs were diluted in Milli-Q water at a ratio of 1:10.

#### 2.5.2. Encapsulation Efficiency (EE)

The NEs were dialyzed (Sigma Aldrich, molecular cut-off of 12,000 Da) for 12 h against Milli-Q water at a ratio of 1:40 (*v*/*v*). As required, 1 mL of dialyzed NEs was taken and mixed with 9 mL of absolute ethanol, followed by a final dilution step with the mobile phase (acetonitrile:0.1% acetic acid (35:65)). The samples were filtered through 0.22 µm membranes and subjected to HPLC (Alliance 2695 equipped with PDA 996 Photodiode Array Detector, Waters, Milford, CT, USA) at a wavelength of 365 nm for the determination of the Qt.

EE [%] = [mass of Qt in the dialyzed NE (mg)/initial mass of Qt added (mg)] × 100

### 2.6. Stability Tests

The stability of the NEs over time at different temperatures, pHs, and NaCl concentrations was evaluated through the changes in the hydrodynamic size, PDI, and zeta potential. The NEs were stored for 30 days at 25 ± 2 °C or 4 ± 1 °C and samples were taken at 1, 7, 14, and 30 days. Their stability at different pH levels (2, 4, 6, 7, and 8) and different concentrations of NaCl (100–500 mM) at pH 6.0 was evaluated after a 6 h incubation period.

### 2.7. Differential Scanning Calorimetry (DSC)

The NEs were previously dialyzed for 12 h and lyophilized (ILSHINBIOBASE, model FD5508, Yangju, Republic of Korea) for 48 h at –48 °C and 20 mTorr. Approximately 4 mg was weighed on an analytical balance (AS 62.R2 PLUS, Radwag, Miami, FL, USA) and sealed in an aluminum container; an empty sealed container was used as a reference. The scan temperature was raised from 20 to 400 °C at a constant rate of 10 °C min^−1^ in a DSC instrument (Perkin-Elmer, model DSC 6000 MT, Norwalk, CT, USA).

### 2.8. Fourier Transform Infrared (ATR–FTIR) Spectroscopy

Before the analysis, the NEs were subjected to the same treatment as described in Section 2.7. Spectral data were collected on an ATR–FTIR spectrophotometer (Agilent Technologies, Cary Model 630, Santa Clara, CA, USA) in the 400–4000 cm^−1^ range, with 20 scans recorded at a <2 cm^−1^ resolution.

### 2.9. Scanning Transmission Electron Microscopy (STEM)

The morphology analysis was performed using a scanning transmission electron microscope (STEM), namely the Inspect F-50 model (FEI, Hillsboro, OR, USA). STEM images were obtained by placing a drop (20 µL) of the NEs on a copper grid (200 mesh, covered with Formvar) (EMS, Philadelphia, PA, USA). Subsequently, the sample was stained with a 1% (*w*/*v*) phosphotungstic acid solution, dried, and visualized on the equipment.

### 2.10. Release Profile in PBS 1X and Krebs Buffer

The quercetin release profile of the previously dialyzed NEs was obtained in an amber container, according to Samadi et al. [20], with slight modifications. First, 2.5 mL of NEs was mixed with 2.5 mL of phosphate-buffered saline (PBS 1X) or Krebs buffer at pH 7.4 and 5.4. Then, it was placed inside a dialysis membrane (12.000 g/mol molecular cut-off) against 95 mL of the buffer solution (PBS or Krebs buffer) containing 20% (*v*/*v*) ethanol. The container was shaken at 120 rpm at 37 ± 0.5 °C. Then, 1 mL of medium was taken at different time intervals (0.5, 1, 2, 4, 6, and 8 h), and the volume was replaced with fresh buffer. Then, the samples were filtered through 0.22 µm membranes, and the Qt was determined using the method described in Section 2.5.2. In the case of the Krebs buffer, the pH (7.4 and 5.4) was maintained during the test using HCl 1.0 M or NaOH 1.0 M, as required.

### 2.11. Release in the Presence of Colonic Bacteria

*Escherichia coli* ATCC 25922: A culture was prepared in Luria–Bertani broth with constant agitation at 37 °C until an optical density (DO600) of 0.7 was reached, equivalent to 5.5 × 10^8^ CFU/mL [21]. A solution was prepared by mixing 0.5 mL of NEs (F1Qt, F4Qt, and P4Qt) with 9.5 mL of the culture medium with and without bacteria (release control) at pH 7.4. Then, the samples were placed inside dialysis bags in front of 90 mL of sterile water. Next, 1 mL was taken at different time intervals (1, 2, 3, 4, 6, and 8.0 h), replacing the culture medium, and the Qt was determined according to the method described in Section 2.5.2. *Bacteroides thetaiotaomicron* ATCC 29741: A culture was prepared in brain–heart broth at 37 °C until an optical density (DO600) of 0.8 was reached, equivalent to 6.0 × 10^8^ CFU/mL [21]. The release assay was performed as previously described for the *E. coli* culture, under anaerobic conditions at pH 7.4.

Similarity tests were performed between the same NE formulations to assess the differences among the release curves obtained with and without bacteria, using the similarity factor (ƒ2), defined as the logarithmic reciprocal square root transformation of the sum of the squared error [22,23]. In this context, ƒ2 values greater than 50 indicate that the two compared curves have a mean difference of no more than 10% at the sample time point, indicating that the profiles are similar.

### 2.12. Evaluation of Cytotoxicity

The cytotoxicity of the NEs was evaluated via the MTT (3-(4,5-dimethylthiazol-2-yl)-2,5-diphenyltetrazolium bromide) assay, as described by Mosmann [24], using the HT-29 cell line. Briefly, 2 × 10^4^ HT-29 cells per well were seeded in 96-well plates and incubated for 24 h at 37 °C with 5% CO_2_. Then, 25 µL of the NE was added at different final concentrations of quercetin (2–40 µM) and the mixtures were incubated for another 24 h. Next, 25 μL of MTT (3 mg/mL) was added and the mixture was incubated for 4 h. Finally, the medium was removed, 100 μL of DMSO was added, and the measurement was performed at 570 nm in a microplate reader (TECAN, Infinite^®^ 200PRO, Männedorf, Switzerland).

### 2.13. Evaluation of Antioxidant Capacity

#### 2.13.1. ABTS and ORAC Assays

The ABTS and ORAC assays were performed according to Intiquilla et al. [25]. The ABTS·+ radical scavenging activity was determined by mixing 200 µL of diluted ABTS·+ solution and 20 µL of PBS (blank), Trolox (standard), or a sample. The absorbance reading was performed at 734 nm after 7 min. The results were expressed as the Trolox equivalent antioxidant capacity (TEAC) and the IC50 value. The oxygen radical absorbance capacity (ORAC) was evaluated at 37 °C in 75 mM phosphate buffer (pH 7.4). The final assay mix (200 µL) contained FL (30 nM), AAPH (12 mM), and an antioxidant (Trolox (0–5 nM)) or sample. The fluorescence reading was performed at 485 and 520 nm of excitation and emission, respectively. It was recorded every 2 min for 120 min in an Infinite M200 Pro plate reader (Tecan Group AG, Männendorf, Switzerland). The ORAC values were expressed as µmol ET/mg Qt.

#### 2.13.2. Antioxidant Capacity in the HT-29 Cell Line

The antioxidant capacity was determined through the dichlorofluorescein diacetate oxidation assay (DCFH-DA), according to Lebel et al. [26], in the HT-29 cell line. Briefly, 12,000 cells/well were added to 96-well plates. After 24 h, the cells were treated with 20 μM of DCFH-DA, in PBS, at 37 °C and 5% CO_2_ for 30 min to allow DCFH-DA to be incorporated into the cell. The wells were then washed with PBS; subsequently, free Qt or an NE loaded with Qt was added and it was allowed to incubate for 6.0 h. Finally, the medium was removed, and 120 μM H_2_O_2_ was added as an inducer of free radical release at the intracellular level. The fluorescence was measured in a Synergy HT multiplate reader (BioTek, Winooski, VT, USA) at excitation and emission wavelengths of 485 nm and 580 m, respectively, at 60 min after adding the inducer.

### 2.14. Statistical Analysis

The experiments were performed in triplicate and results were reported as the mean ± standard deviation. Data were analyzed using GraphPad Prism 6 (GraphPad Software, San Diego, CA, USA) and Origin 8.0 (Origin Lab, Northampton, MA, USA). A one-way analysis of variance (ANOVA) was conducted, and the Tukey test was used for the multiple comparison of means with a significance level α = 0.05 (95% confidence).

## 3. Results and Discussion

### 3.1. Isolation of Chenopodin

**Figure 1:**
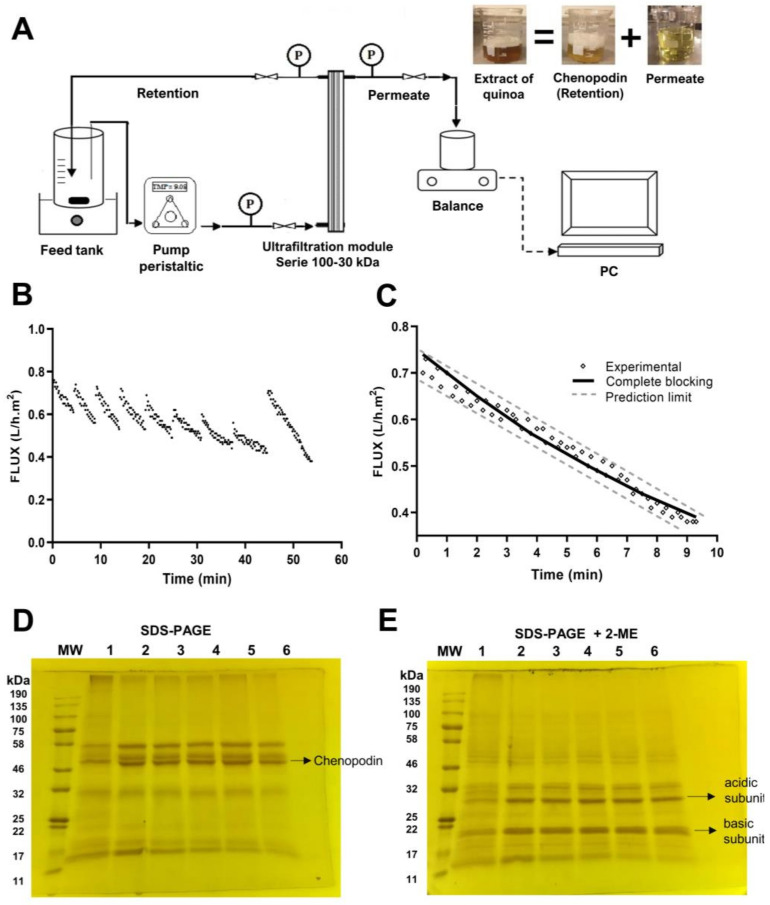
Chenopodin (QP) isolation process. (**A**). Work scheme developed in the crossflow ultrafiltration (UF) system. (**B**). Flux vs. time graphs of the diafiltration (DF100-30) process and concentration (UFC100-30). (**C**). Mathematical modeling of the membrane fouling process in the UFC100-30 process. (**D**). SDS-PAGE electrophoretic profile under non-reducing and (**E**) reducing conditions using β-mercaptoethanol (2-ME). Mw is the molecular weight marker. 1. Quinoa extract, 2. 2DF, 3. 4DF, 4. 6DF, 5. 8DF, and 6. UF_C_100-30.

In previous work, Arazo et al. [16] developed a discontinuous DF/UF crossflow filtration procedure to obtain chenopodin (method 1), which was modified to reduce the operation time and increase the protein yield (method 2, this research). Table 1 shows the results for both methods. The modifications concerning method 1 were the following: (i) both 30 and 100 kDa membranes were used together for the diafiltration (DF) and concentration (UFc) steps and (ii) the sequence of use of the membranes was reversed, i.e., the 100 kDa membrane was placed first in the feed direction, followed by the 30 kDa membrane (DF100-30 and UFc100-30)—see Figure 1A. The change in the membrane sequence was implemented because a decrease in the permeate flux from 0.72 to 0.58 L/h.m^2^ was evident when the membrane sequence was 30 kDa followed by 100 kDa, increasing the processing time.

Figure 1B shows the flow versus time graph of DF100-30 + UFc100-30 for method 2. DF100-30 was used at a TMP of 9.72 kPa and a ratio of extract/buffer of 1:1 *v*/*v* to eliminate impurities (small proteins, amino acids, salts, and others). Method 2 reduced the DF time by 42.43% compared to method 1—see Table 1. The simultaneous use of the 100 and 30 kDa membranes (DF100-30) presented higher permeate flux (0.40 L/hm^2^ after the eight diavolumes) compared to method 1 (0.26 L/hm^2^) using only the 30 kDa membrane independently. The lower permeate flux was due to the decreased pore size and the concentration polarization layer that formed on the membrane surface, which became thicker during the process, causing lower permeation and high membrane resistance [27]. The concentration process (UFc) at the same TMP (9.72 kDa) in method 2 allowed a higher permeate flux (0.72 L/hm^2^) compared to method 1 (0.55 L/hm^2^). The diafiltered quinoa protein extract (DF100-30) concentrated by UFc100-30 yielded 2.14 g of QP/400 mL, which was significantly higher (0.884 g of QP/200 mL) than in method 1. The protein yield increased from 26.14% (*w*/*w*) (method 1) to 32.75% (*w*/*w*) (method 2)—see Table 1. The membrane fouling mechanism was observed as complete pore blockage (R^2^_adj_ = 0.993), according to the Hermia model [19]—see Figure 1C. Arazo et al. [16] obtained the same result with method 1.

The integrity of the proteins isolated through method 2 was analyzed via SDS-PAGE electrophoresis under non-reducing (Figure 1D) and reducing (Figure 1E) conditions. Samples were taken during the use of DF100-30 (2, 4, 6, and 8 diafiltrations) and UFc100-30. Under non-reducing conditions, the leading bands of 50–57 kDa (red line) were seen in DF100-30 and with even greater intensity in UFc100-30, corresponding to quinoa 11S globulin (QP), similar to the results reported by Arazo et al. [16]. A band of 31 kDa corresponded to the acid subunit of QP, and another of ~12 kDa was related to albumin 2S, which decreased in intensity until it almost disappeared during the process of DF100-30 + UFc100-30 [16,28]. Under reducing conditions, the 54–57 kDa bands connected by disulfide bonds were disrupted in the presence of 2-β-mercaptoethanol (2-ME), giving rise to intense bands corresponding to the acidic (31 kDa) and basic (21 and 23 kDa) states of QP [29]. In conclusion, method 2 improved the QP isolation process, reduced the operation time, and gave a higher yield and a profile of protein bands in SDS-PAGE electrophoresis, similar to method 1. Therefore, method 2 was used for the isolation of chenopodin in this study.

### 3.2. Preparation of Chenopodin/Alginate Soluble Complex (QPA)

In a previous work, Romo et al. [15] used QP isolated via isoelectric precipitation to prepare soluble QP/alginate (QPA) complexes in ratios of 4:1, 3:2, 1:1, 2:3, and 1:4, respectively. Circular dichroism (CD) analysis indicated a decrease in the content of α-helices and an increase in β-sheets in the secondary structure of QP, as well as a reduction in the surface hydrophobicity (S_0_) of the QPA complex when the ratio of QP decreased, possibly due to interactions with alginate. ATR–FTIR confirmed the intermolecular interaction between QP and Alg via hydrogen bonding for the ratios of 3:2, 1:1, and 2:3, as evidenced by the shift and decrease in a band from 3280 to 3284 cm^−1^. Moreover, a hydrophobic interaction was evidenced by the red shift of amide II (1541 cm^−1^) and a reduction in the amide I band (1634 cm^−1^). Additionally, an electrostatic interaction was identified at all ratios when the amide I peak of QP coupled with the strong absorption band of the carboxylate (alginate) at 1409 cm^−1^ and shifted to lower wavelengths.

Arazo et al. [17] prepared the same soluble QPA complexes but used QP isolated via crossflow DF/UF filtration (method 1—see Section 3.1). The QP/Alg ratios of 4:1 and 3:2 presented smaller sizes (<120 nm) and a greater intensity of S_0_, i.e., greater surface hydrophobicity. The CD analysis showed that the 3:2 ratio of QPA presented fewer α-helix structures (17.50%) and more β-sheet structures (35.35%) than other proportions, favoring the exposure of hydrophobic amino acids (aromatic and aliphatic) on the surface of the protein, which can interact with alginate in the formation of the QPA complex [30]. Additionally, this proportion presented higher thermal stability in the DSC, TGA, and intermolecular interaction mechanisms regarding the hydrogen bonds and hydrophobic and electrostatic interactions between the QP and alginate, in line with those described by Romo et al. [15]. The characteristics presented suggest that QPA (3:2) could be a valuable natural surfactant for the development of emulsions due to the presence of hydrophobic regions [31].

**Figure 2:**
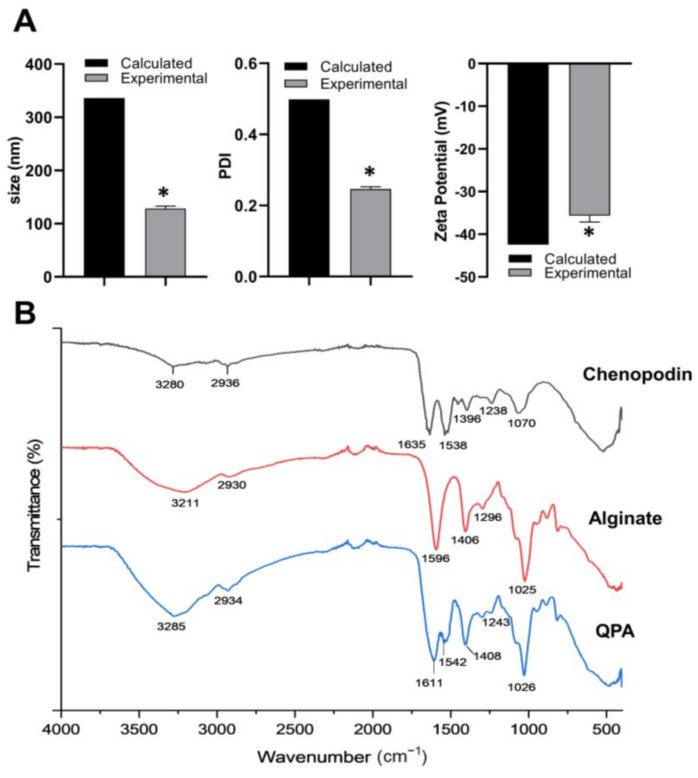
Characterization of the soluble complex QPA. (**A**). Size, PDI, and zeta potential calculated and experimental values. (**B**). ATR-FTIR spectra of chenopodin (QP), alginate, and QPA complex. * Significant difference between calculated and experimental values (*p* < 0.05).

In this work, the soluble complex QPA (3:2) was prepared using QP isolated via method 2. The formation of the protein/polysaccharide complex was confirmed by comparing the values of the size, PDI, and zeta potential obtained from the experimental data with the calculated values obtained from the weighted sum of the mass fraction of QP and alginate via the individual values of each measured property (Figure S1) [15]. Figure 2A shows the experimental values of the soluble QPA complex (3:2) in terms of size (130.9 nm), PDI (0.246), and zeta potential (−35.9 mV). They are significantly lower (*p* < 0.05) than the calculated values, which indicates an interaction between the biopolymers that causes the formation of a more ordered structure, with intermolecular interactions that favor their stability [15,32].

Figure 2B shows the ATR–FTIR spectra of QP, alginate, and the QPA complex. The QPA complex shows an increase in and the displacement of the band corresponding to the tension vibration of the N-H and O-H groups from 3280 to 3285 cm^−1^, showing the formation of intermolecular hydrogen bonds between the biopolymers [33]. The band corresponding to amide I of QP at 1635 cm^−1^ was coupled with the strong band related to the asymmetric stretching of the alginate carboxylate, causing the displacement of the amide I band at 1611 cm^−1^, which indicated an electrostatic interaction between the negatively charged carboxylate group and positively charged areas or “patches” of the protein, probably with the side chains of amino acids such as arginine, lysine, and histidine [15,32,34]. The amide II band was also slightly shifted from 1538 to 1542 cm^−1^, characteristic of the conformational change from the α-helix type to a more organized β-sheet structure in the protein, which is evidence of a hydrophobic interaction [30,35]. These interaction mechanisms were the same as those observed by Romo et al. [15] and Arazo et al. [17].

### 3.3. Preparation and Characterization of NEs

The NEs were prepared using Tw80 (F1) as the only surfactant and it was partially replaced with the QPA complex (3:2) in QPA/Tw80 at ratios of 20:80 (F2), 40:60 (F3), 60:40 (F4), 70:30 (F5), and 80:20 (F6) *w*/*w*, respectively—see Table 2. F1 was used as the comparison reference in this study. F1 was obtained from the NEs developed by Robledo et al. [12], using Tw80 at 10% (*w*/*w*). The percentage of Tw80 was reduced to 2.0% (*w*/*w*) using a high-energy methodology (ultrasound) to decrease its content and avoid exceeding its acceptable daily intake (ADI) value of 10 mg/kg/day in the formulations [36]. F1 presented a size of 170.7 nm, PDI of 0.215, and zeta potential of −5.67 mV. The reduced size and polydispersity can be explained by the fact that Tw80 has a small size (1.23 kDa), allowing its rapid adsorption in the forming nanodroplets, quickly stabilizing them via steric repulsion [37]. The negative zeta potential presented was due to the imbalance of the ions produced by the water itself (i.e., H^+^ and OH^−^) at the aqueous interface, inducing the preferential adsorption of OH- ions on the surface of the NE [38].

F2 to F6 (without Qt) showed an increase in size from 178.3 to 263.0 nm, in PDI from 0.241 to 0.252, and in zeta potential from −7.97 to −14.53 mV, depending on the proportions of the QPA complex (20–80% *w*/*w*) in the QPA/Tw80 surfactant system. The increase in size occurred because QPA is composed of QP (54–57 kDa) and alginate (162 kDa), polymers with a larger molecular size than Tw80, slowing down their adsorption on the nanodroplets, generating a variety of sizes, and increasing the value of the PDI [15,37]. The NEs prepared with the QPA/Tw80 surfactant system (F2 to F6) were smaller than the theoretical (additive) sizes calculated for F2 (445 nm), F3 (719 nm), F4 (994 nm), F5 (1131 nm), and F6 (1268 nm). Likewise, they presented higher PDI values than those calculated (0.223 to 0.247), showing the molecular rearrangement of QPA in the formation of the NEs [15]. The zeta potential showed an increase in negative charge when the proportion of QPA in the formulation increased, showing that the QPA complex covered the oily phase of the NE. The experimental values of the zeta potential obtained were of lower intensity than the calculated values (additive) for F2 (−11.1 mV), F3 (−16.5 mV), F4 (−21.8 mV), F5 (−24.5 mV), and F6 (−27.2 mV)—see Table 2. The lower values of the zeta potential were due to a molecular rearrangement that favored the more significant electrostatic interaction between the positive patches of chenopodin exposed towards the aqueous phase and the alginate, reducing the net negative charge around the NE [15,17]. F2 and F3 presented a lower intensity in terms of the zeta potential due to the more significant presence (>50%) of Tw80. F5 to F6 showed significant differences at *p* < 0.05 concerning F1 due to the presence of QPA (>60) in the surfactant system.

The NEs loaded with Qt (F1Qt to F6Qt) presented significant differences (*p* < 0.05) in the studied parameters concerning their NE controls (without Qt)—see Table 2. F1Qt showed a slight decrease in size at 160.1 nm and an increase in PDI at 0.232, indicating a molecular rearrangement of F1 in the presence of Qt. The 7-OH of the resorcinol residue, the 4′-OH of the catechol residue, and the carbonyl group of the Qt ring formed hydrogen bonds with the (polar) polyoxyethylene region of Tw80. In addition, a hydrophobic interaction with the monooleate (apolar) of the surfactant and the carbon chains of MCT in the nucleus generated changes in size and PDI. A similar effect was described by Mendoza-Wilson et al. [39] when encapsulating Qt in an NE stabilized only with Tween 65.

F2Qt to F6Qt presented an increase (*p* < 0.05) in size and PDI compared to their control NEs (without Qt) due to the additional effect of Qt on the NE. Qt can interact with QPA via different interaction mechanisms (hydrogen bonding, hydrophobic, and van der Waals) to achieve its inclusion in the nanodroplet, causing molecular rearrangements [39]. The increase in the zeta potential was due to the restructuring of the QPA complex in the presence of Qt, favoring the exposure of negative patches of QP and alginate to the interfacial zone of the NE, which were negatively charged because they were above its pI (4.50) and pKa (3–3.5), respectively [15,17]. F2Qt and F3Qt presented the lowest zeta potential intensities of −18.33 and −19.0 mV compared to the other NEs due to the lower presence of QPA (20 and 40%, respectively) in the QPA/Tw80 surfactant system. F4Qt to F6Qt (>50% QPA) presented a negative zeta potential of less than −20 mV, which was necessary to favor the repulsion between nanodroplets, improving their dispersion and stability [40].

F1Qt presented a high EE values of 90.78% of Qt, due to the small size and rapid adsorption of Tw80 on the surfaces of the nanodroplets, favoring the encapsulation of Qt [37]. F2Qt to F6Qt, stabilized by the QPA/Tw80 surfactant system, did not show significant differences (*p* < 0.05) compared to F1Qt, maintaining EE values above 85%—see Table 2. The surfactant power of QPA would be favored by the high surface hydrophobicity of the QPA complex at the 3:2 ratio, as previously determined by Romo et al. [15] and Arazo et al. [17]. Additionally, it has been determined that the primary structure of QP (Q6Q385, UniProt) has Domain I (206 aa) and Domain II (150 aa) in its polypeptide chain, which has 32 and 44% aa of apolar nature, respectively, favoring the interaction with Qt [15,41]. The EE values achieved by the F2Qt to F6Qt NEs were similar to those obtained in other works that used protein/polysaccharide surfactant systems to encapsulate hydrophobic compounds, such as that of López-Monterrubio et al. [14], who used a soluble whey protein/high methoxyl pectin complex (WPH-HMP) to encapsulate between 86.80 and 91.99% β-carotene. To date, no studies have been reported using proteins of plant origin in protein/polysaccharide complexes as NE surfactant systems to encapsulate Qt.

The loading capacity (LC) of the NEs was directly related to the concentration (mg/mL) of Qt encapsulated in the NEs. The concentration of Qt in all the NEs was higher than 0.86 mg/mL. This work used 25 mg of Qt in a total volume (oily phase + aqueous phase) of 25 mL. Higher concentrations of Qt (50 and 100 mg) were evaluated in the NEs, but they resulted in EE of less than 50%. The amount of net Qt per formulation was ~21 mg, which could be delivered in lyophilized form in two doses per day, sufficient to exert curative effects against inflammatory processes. Li et al. [42] reported that doses of 2 to 10 mg of Qt in rats (200–250 g of weight) were effective in inhibiting proinflammatory mediators (IL-1β, IL-6, TNF-α).

In summary, the NEs that used the QPA/Tw80 surfactant system (F2Qt to F6Qt) presented sizes smaller than 350 nm and PDI < 0.3. The QPA complex maintained EE above 85%, reducing the use of Tw80.

### 3.4. Stability of NEs at Different Times, Temperatures, pH Levels, and NaCl Concentrations

**Figure 3:**
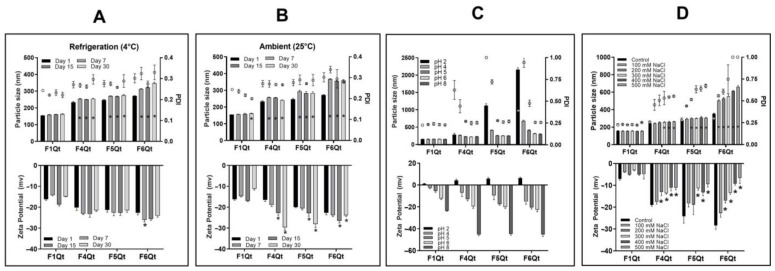
Size, PDI, and zeta potential of NEs F1Qt, F4Qt, F5Qt, and F6Qt in storage at (**A**). refrigerated temperatures (4 °C) at pH 6.0, (**B**). ambient temperatures (25 °C) at pH 6.0, (**C**). different pH values (2.0–8.0) at 25 °C, and (**D**). different NaCl concentrations (100–500 mM) at pH 6.0 and 25 °C. * indicates *p* < 0.05 vs. day 1 or NE control (without NaCl).

The stability of F1Qt, F4Qt, F5Qt, and F6Qt was evaluated under different storage conditions, determining changes in size, PDI, and zeta potential. The performance of the NEs was evaluated at room temperature (25.0 °C) and under refrigeration (4.0 °C) at 1, 7, 15, and 30 days of storage. F1Qt at refrigeration temperatures did not show significant changes (*p* < 0.05) until day 30, maintaining a size of 162.5 nm, PDI of 0.22, and zeta potential of −14.9 mV—see Figure 3A. At 25 °C, on day 30, the size increased to 168.1 nm, but it maintained its polydispersity (0.20) and zeta potential (−14 mV)—see Figure 3B. The minimal changes in F1Qt regarding the studied parameters were due to the high chemical stability of Tw80 as a surfactant, favored by the steric repulsion stability mechanism that exists between the nanodroplets, due to the nonionic nature of Tw80 [14,43].

At 4.0 °C, F4Qt, F5Qt, and F6Qt significantly increased (*p* < 0.05) in size from day seven but remained at a nanometric scale until day 30 (<10% compared to day 1). They presented maximum sizes of 253.9, 274.7, and 346.6 nm, respectively. The PDI and zeta potential did not change until day 30. At a temperature of 25 °C, F4Qt, F5Qt, and F6Qt showed an increase in size of more than 15% until day 30, with maximum values of 255.1, 289.6, and 365.9 nm, respectively. The increase in size compared to storage at 4.0 °C occurred because the Ostwald ripening process is favored at higher temperatures than refrigeration [9,44]. An increase in temperature causes droplet collision, where larger droplets grow at the expense of smaller ones due to their greater molecular diffusion through the continuous phase [40]. The PDIs of F4Qt and F5Qt did not change, but they showed an increase in the intensity of the negative charge of up to −29.7 and −27.1 mV. The change in the zeta potential may have occurred because the biopolymers adsorbed at the oil/water interfaces of the NEs increased on the surface, causing the rearrangement of the QPA complex, favoring a more significant negative charge in the NEs [9]. F6Qt presented the largest increase in PDI to 0.35 on day 30, presenting physical instability, which was correlated with the lower intensity of the zeta potential compared to F4Qt and F5Qt, despite containing a greater proportion of QPA in the surfactant system.

The effect of different pH values (2.0 to 8.0) on the NEs was evaluated. F1Qt showed minimal changes in size (159.4 nm), PDI (0.234), and zeta potential in the studied pH range. At pH 8.0, it only showed a significant increase (*p* < 0.05) in zeta potential at −23.1 mV, possibly due to the strong adsorption of OH on the NE surface after adjusting the pH [38]. The minor changes in F1Qt at different pH values (2.0 to 8.0) were due to Tw80 being a nonionic surfactant that stabilizes NEs via steric repulsion, making it stable under changes in pH and ionic strength [45]. F4Qt, F5Qt, and F6Qt presented significant changes (*p* < 0.05) in size and PDI at pH< 6.0 (starting pH). At pH 2, the largest sizes of 286.9, 1122, and 2158 nm and PDIs of 0.629, 1.0, and 0.945, respectively, were seen. The alterations in size and PDI occurred due to the loss of solubility of the QPA complex because it was at a pH (2–5) close to the pI (4.5) of QP or lower than the pKa (pH 3.0–3.5) of alginate, reducing the solubility of the polymers, generating weak electrostatic repulsion between the nanodroplets, and promoting their aggregation [15,46]. At pH 2, the zeta potential shifted to positive values, namely +4.3, +5.8, and +6.5 mV (see Figure 3C), due to the presence of QP in F4Qt, F5Qt, and F6Qt, respectively. Romo et al. [15] estimated the charges of Domain I (aas 42–247) and Domain II (aas 305–454) that represented QP (480 aas) at pH 2.0, obtaining values of +54 and +36, respectively. However, these are partially neutralized by the electrostatic interaction with the alginate, reducing the net charge of the QPA that remains on the surface of the NEs [40]. F4Qt showed better stability than F5Qt and F6Qt, possibly due to the high proportion of Tween 80 (40%) in the QPA/Tw80 surfactant system.

At pH 8.0, F4Qt, F5Qt, and F6Qt did not present significant differences (*p* < 0.05) in size and PDI concerning the NEs prepared at pH 6.0. However, a significant increase (*p* < 0.01) in the intensity of the zeta potential was evident at −42.5, −44.7, and −46.5 mV, respectively. Estimating the charges of the patches present in Domain I (−6) and Domain II (+4) of QP, these presented a total net negative charge (−2) at pH 8.0. Furthermore, the carboxylic functional group (R-COO-) of alginate would be up to 100 times more ionized than at pH 6.0, explaining the increase in the negative charge [15,47].

The NEs were evaluated at different NaCl concentrations (100 to 500 mM) at pH 6.0. F1Qt did not present significant changes (*p* < 0.05) in the studied parameters at the different NaCl concentrations due to the nonionic nature of Tw80 (Figure 3D). F4Qt and F5Qt maintained a stable size at 100 mM NaCl, similar to the physiological concentration of NaCl (117 mM). However, they began destabilizing (*p* < 0.05) at 200 mM NaCl, increasing in size to 254.3 and 302.2 nm, respectively. At 100 mM NaCl, F6Qt showed a significant increase (*p* < 0.01) in size (505 nm). Likewise, the PDI increased significantly (*p* < 0.5) at 100 mM NaCl for all formulations, but with greater intensity (PDI > 0.5) at concentrations higher than 200 mM NaCl. Finally, F4Qt, F5Qt, and F6Qt showed a decrease in the intensity of the zeta potential depending on the increase in the salt concentration, reaching lower intensity values of −11.1, −9.4, and −6.6 mV at 500 mM NaCl, respectively. These results show that the destabilization of the NEs occurred mainly due to the shielding of the QPA surfactant charges by the high concentration of NaCl (200–500 mM). This first decreases the interaction between QP and alginate, destabilizing the QPA surfactant. Secondly, it reduces the surface charge of the NEs, which reduces the electrostatic repulsion between the nanodroplets, promoting the predominance of van der Waals attractive forces, which generate aggregation and flocculation [48]. This increase in the ionic strength correlates with the increase in size and PDI, indicating that the QPA complex covers the oil droplets, stabilizing them via an electrostatic repulsion mechanism [49]. Bather et al. [50] described similar behavior in gelatin/alginate complexes (3:1 *w*/*w*) in NaCl (50–200 mM), decreasing the electrostatic interaction between the polymers and screening the residual charge of the complex later due to the increase in ionic strength. This effect ultimately leads to conformational changes in the secondary structure of the protein, increasing random coil structures and decreasing the α-helix conformation, favoring the formation of short-distance hydrogen bonds to keep the complex stable.

In summary, F4Qt and F5Qt are more stable, and further characterization and performance tests can be developed with these formulations. Additionally, to elucidate the role of QP, two new formulations, P4Qt and P5Qt, were added, where the QPA complex was replaced with QP. The size, PDI, zeta potential, EE, and LC of P4Qt and P5Qt were 222.2 and 248.6 nm, 0.257 and 0.259, −23.0 and −25.7 mV, 92.27 and 87.10%, and 0.92 and 0.87 mg/mL, respectively.

### 3.5. DSC Analysis

**Figure 4:**
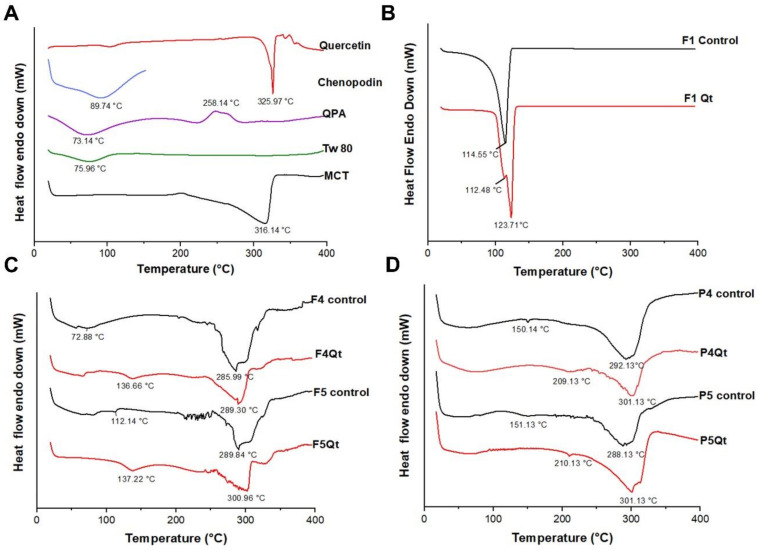
DSC profiles of (**A**). Qt, QP, QPA, Tw80, and MCT; (**B**). F1 control and F1-Qt; (**C**). F4 control, F4-Qt, F5 control, and F5-Qt; (**D**). P4 control, P4-Qt, P5 control, and P5-Qt.

Figure 4A shows the DSC analysis of the components of the NEs, NE controls (without Qt), and NEs loaded with Qt (NE-Qt). Quercetin presented endothermic peaks at 103.14 and 325.97 °C that corresponded to the dehydration and melting point of the flavonoid [51]. QP showed an endothermic peak at 89.74 °C, related to the temperature of its denaturation. QPA showed an endothermic peak at 75.14 °C and an exothermic peak at 248.14 °C, corresponding to the loss of water associated with the biopolymers and their degradation, respectively [15,17]. MCT showed an exothermic peak at 316.14 °C, associated with its decomposition.

The F1 control and F1Qt were analyzed in liquid form due to the oily nature of their components. The F1 control presented an endothermic peak at 114.55 °C, related to the loss of water, which constituted 90% of the sample. In comparison, in F1Qt, the endothermic peak moved to 123.71 °C—see Figure 4B. Peak displacement at higher temperatures shows an intermolecular interaction between Qt and Tween 80 [52]. This behavior was also observed by Zhang et al. [53] in NEs stabilized with Tw80, shifting the endothermic peak from 111.9 °C (NE control) to 127.1 °C (NE-Vitamin D3); this was due to electrostatic and hydrophobic interactions and hydrogen bonds between Vitamin D3 and Tw80, as evidenced in the FTIR analysis.

The F4 and F5 controls presented endothermic peaks at 72.88 and 112.14 °C, respectively (Figure 4C). These peaks were related to the elimination of residual water bound to the QPA complex [54]. The F5 control, with a larger proportion (70%) of the QPA complex in the QPA/Tw80 surfactant system, showed higher levels of water retention, which caused the displacement of the endothermic peak to higher temperatures compared to the F4 control [15]. Zhou et al. [9] reported a similar profile when stabilizing NEs with a soy protein/alginate complex (2%/0.02–0.1%), where endothermic peaks between 122 and 131 °C were evidenced due to the electrostatic interaction and hydrogen bonds that existed between the protein/polysaccharide complex and MCTs. Additionally, the F4 and F5 controls showed peaks at 285.99 and 289.30 °C related to the degradation of MCTs (316.14 °C), the main components (∼50%) of the lyophilized NEs. The decrease in the degradation temperature was due to the presence of free water in the formulations. In the presence of the flavonoid, F4Qt and F5Qt presented an endothermic peak that shifted to higher temperatures of 136.66 and 137.32 °C, respectively. The presence of Qt caused the higher thermal stability of the NEs because it favored the greater affinity of the components of the QPA/Tw80 surfactant system for water, stabilizing the protein that formed the complex [17]. The characteristic MCT peak shifted from 285.99 to 289.30 °C and from 289.84 to 300.96 °C, respectively, which may have indicated the dispersion of Qt in the oil phase, promoted by intermolecular interactions such as hydrogen and hydrophobic bonds [55].

The P4 and P5 controls presented endothermic peaks at 150.14 and 151.13 °C, respectively. The endothermic peaks were higher compared to those reported for the NEs stabilized by the QPA/Tw80 surfactant system, which was related to the strong binding of water with QP in the presence of Tw80 and MCTs. Jin et al. [56] reported a similar profile for NEs stabilized with soy protein/Tween20 and whey protein/Tween 20 complexes, presenting endothermic peaks at 164.41 and 167.25 °C, respectively. The increase in the thermal stability of the proteins was a result of the electrostatic interaction between the protein and the nonionic surfactant. In the presence of Qt, P4Qt and P5Qt showed a peak shift of 209.13 and 210.13 °C, respectively, indicating a possible interaction of Qt with the QP/Tw80 surfactant system, showing the importance of QP in Qt encapsulation (Figure 4D). In summary, the characteristic endothermic peaks of the NE controls showed increased temperatures in the presence of Qt, indicating greater thermal stability. The absence of the distinct fusion peak of Qt stands out, suggesting its molecular dispersion in the nanodroplets, changing from a crystalline state to a high-energy amorphous state, and being trapped and protected by the surfactant system [57,58].

### 3.6. ATR–FTIR Analysis

**Figure 5:**
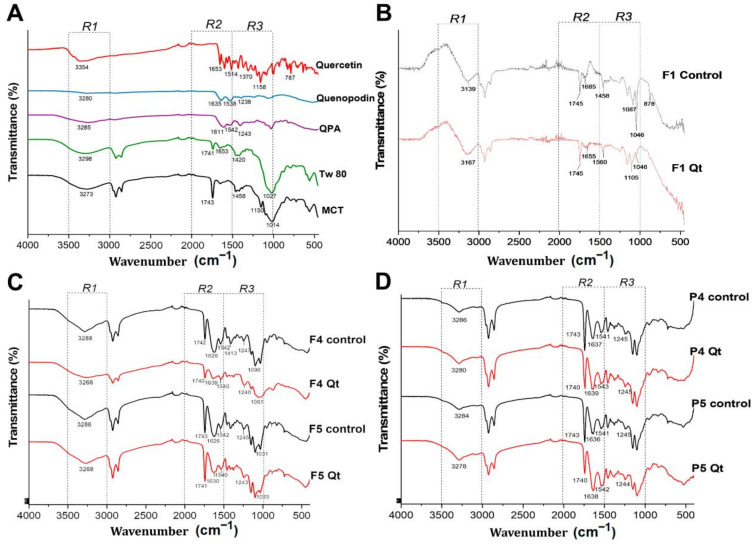
ATR–FTIR profiles of (**A**). Qt, QP, QPA, Tw80, and MCT; (**B**). F1 control and F1-Qt; (**C**). F4 control, F4-Qt, F5 control, and F5-Qt; (**D**). F4 control, F4-Qt, F5 control, and F5-Qt.

The ATR–FTIR spectra showed the interactions between the different components of the NEs. The spectrum was separated into three regions (R1-R3) for a better understanding. Firstly, the profile of the individual components of the formulation is presented in Figure 5A. Tw80 presented bands at 3298 cm^−1^ corresponding to O-H stretching, at 1653 cm^−1^ for C=C stretching, and at 1458 cm^−1^ for the CH_2_ stretching of the carbon chain, as well as a broad band at 1027 cm^−1^ indicating the C–O stretching of the ether and ester groups of the ring and hydrocarbon chain of the surfactant [59]. At 1743 cm^−1^, MCT presented the stretching of C=O; at 1458 and 1380 cm^−1^, it showed the stretching of CH_2_ present in the length of the hydrocarbon chain and the stretching of the C-H group in the methyl terminal, respectively [9]. Quercetin showed a prominent band at 3354 cm^−1^ that corresponded to O-H stretching vibrations and at 1653 cm^−1^ corresponding to the asymmetric bending vibration of C=O. The bending of aromatic groups was associated with the bands at 1514, 1564, and 1599 cm^−1^; the phenolic -OH bending of the aromatic ring was seen between 1241 and 1370 cm^−1^, as well as asymmetric (C–CO) bending at 1241 cm^−1^, C-H bending at 1360 cm^−1^, C-H in-plane bending at 1309 cm^−1^, and out-of-plane bending between 999 and 600 cm^−1^ for aromatic hydrocarbons [17]. The findings for QP and the QPA complex are described in Figure 2B in Section 3.2.

Figure 5B shows the profile of the F1 control and F1Qt. The R1 region shows a band at 3139 cm^−1^ corresponding to O-H stretching, which, after adding Qt (F1Qt), shifted to 3167 cm^−1^. Likewise, in R3, there was a substantial reduction in the 1046 cm^−1^ band, corresponding to the C-O stretching of the ether and ester groups of the structure of the sorbitan monoleate of Tw80, indicating a hydrogen bond interaction between the oxygen of the surfactant and the OH group of Qt [52]. In R2, the characteristic band of the C=C stretching of the Tw80 was shifted at 1655 cm^−1^. R3 showed a band shift at 1105 cm^−1^ for C-O vibration and a diminished signal at 878 cm^−1^, corresponding to the C-H bending of the hydrocarbon chain of the surfactant, showing a hydrophobic interaction between Qt and Tw80. This confirms the molecular dispersion of Qt in the nanodroplet, as evidenced in the DSC analysis [59].

Figure 5C shows the formulations stabilized by QPA/Tw80 with and without Qt. In R1, the F4 control (without Qt) presented a strong band at 3288 cm^−1^ corresponding to N-H and O-H stretching. After adding Qt (F4Qt), the displacement and greater amplitude of the band were seen at 3268 cm^−1^, indicating a hydrogen bond interaction between Qt and QPA/Tw80. It has been stated that protein/polysaccharide complexes can form hydrogen bond interactions with flavonoids, resulting in a significant increase in intensity and/or bandwidth in the region of 3200–3400 cm^−1^ [55,60]. In the R2 region, the F4 control presented bands at 1740 cm^−1^ for C=O stretching, at 1626 cm−1 for amide I, at 1540 cm^−1^ for amide II, and at 1247 cm^−1^ for amide III, related to the protein present in QPA/Tw80. F4Qt showed a decrease in, and the displacement of, amide I at 1639 cm^−1^, as well as a decrease and slight red shift in amide II at 1542 cm^−1^, showing a hydrophobic interaction between Qt and QPA/Tw80 [15]. Furthermore, the band at 1639 cm^−1^ indicated the majority β-sheet conformation of the QP, which favors the unfolding of the protein and the exposure of hydrophobic amino acids such as leucine, alanine, valine, and phenylalanine, in turn favoring the interaction with Qt [15,61]. This result coincides with the high surface hydrophobicity of the soluble QP/alginate complex (3:2) described by Arazo et al. [17]. The F5 control and F5Qt presented the same profile and behavior as the F4 control and F4Qt, respectively.

Figure 5D shows the NEs stabilized by the QP/Tw80 surfactant system with and without Qt. In R1, the P4 control presented a band at 3286 cm^−1^ related to the stretching of the O-H and N-H of QP, which then, in P4Qt, increased and shifted to 3280 cm^−1^, showing a hydrogen bond interaction between Qt and QP/Tw80. In R2, a red shift in amides I and II was observed at 1639 and 1543 cm^−1^, respectively, indicating hydrophobic interactions between the flavonoid and QP [15]. P5Qt showed similar spectra. The changes in P4Qt and P5Qt were similar to those in the NEs stabilized by QPA/Tw80, highlighting QP’s critical role as a surfactant agent in the QPA complex.

In summary, in the R1 region, the formation of hydrogen bonds was observed, possibly between the OH group of the aromatic ring of Qt and the -NH and -OH groups of QP present in the QPA/Tw80 and QP/Tw80 surfactant systems. In R2 and R3, the hydrophobic interactions due to the decrease in and displacement of amides I and II of QP coincide with the results reported in other investigations [55,62,63]. Finally, the encapsulation of Qt is confirmed by the absence of the characteristic bands of the aromatic ring of Qt (1514, 1564, and 1599 cm^−1^) in the analysis.

### 3.7. STEM Images of NEs

**Figure 6:**
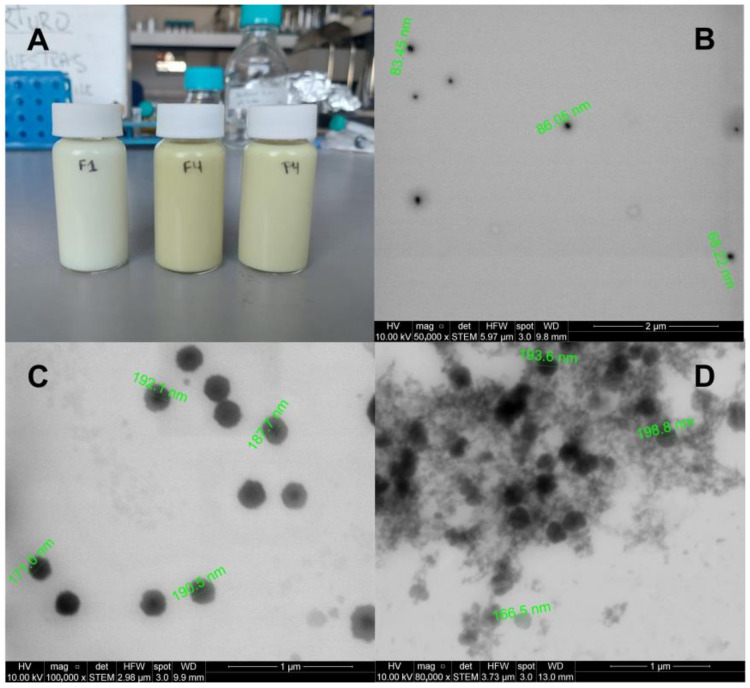
(**A**). NEs F1-Qt, F4-Qt, and P4-Qt and STEM images of (**B**) F1-Qt, (**C**) F4-Qt, and (**D**) P4-Qt.

Figure 6 shows the visual appearance (A) of the NEs. F4Qt and P4Qt are yellow due to the higher concentrations of Qt and the presence of the QP/Al complex compared to F1Qt. Figure 6B–D show the STEM images of F1Qt, F4Qt, and P4Qt, respectively. Figure 6B shows F1Qt, which appears as a spherical nanodroplet with a dark center (MCT) and a transparent halo, corresponding to the hydrophilic layer that surrounds the oil phase, a product of the hydrophilicity of Tw80 (polyoxyethylene esters) [53]. F4Qt (see Figure 6C) presents a dark core surrounded by an opaque halo that is 40 to 60 nm thick—this corresponds to the soluble QPA complex, which adsorbs water around it, favoring the stability of the nanodroplet and increasing its size in comparison to F1Qt. This effect of the opaque halo was also evident in an NE stabilized by a whey protein/chitosan complex [64]. On the other hand, P4Qt (see Figure 6D) presents nanodroplets with a dark halo that is difficult to differentiate from the core and an abundant gelled QP network in the form of “string clusters”. From the STEM images, it is clear that the QPA complex (F4Qt) produces a more ordered nanostructure compared to QP (P4Qt). The average sizes for F1Qt, F4Qt, and P4Qt were 85.2 ± 15.1, 205.8 ± 18.2, and 192.9 ± 21.2 nm, respectively. These values were lower than those measured by the Zetasizer, because TEM measures dry samples, while the Zetasizer determines the sizes of samples dispersed in water via the DLS method, based on the determination of the Brownian motion of the particles and its subsequent extrapolation to hydrodynamic sizes using the Stokes–Einstein equation [21].

### 3.8. Dissolution Studies in Simulated Colonic Media

#### 3.8.1. Comparison between Compendial (PBS1X) and Biorelevant (Krebs) Buffers

The release profile of Qt was evaluated in the PBS1X and Krebs buffers, both at pH 7.4 (healthy colon) and 5.4 (diseased colon). The expected maximum concentration of Qt (Cmax) in the release medium corresponded to 0.023 mg/mL; therefore, sink conditions were not achieved in the pH 7.4 and 5.4 buffers, where the solubility has been reported to be 0.002 mg/mL at pH 5.0 and 0.013 mg/mL at pH 7.0 [65]. Then, 20% ethanol was added to the receiving phase in each medium to obtain sink conditions, where the reported solubility was greater than 0.96 mg/mL [66], equivalent to 42 times the Cmax.

**Figure 7:**
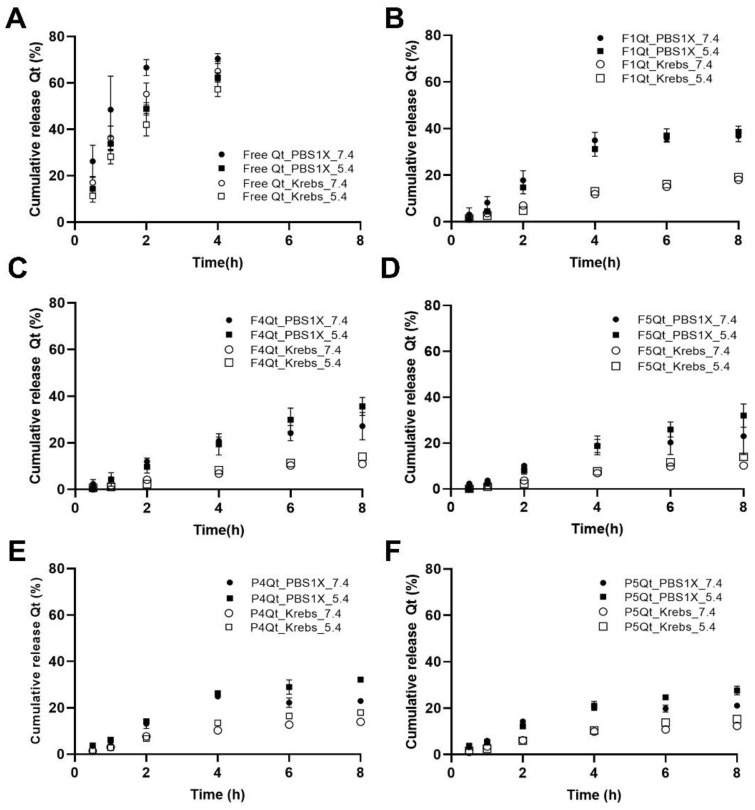
Release profiles of (**A**). free Qt, (**B**). F1Qt, (**C**). F4Qt, (**D**). F5Qt, (**E**). P4Qt, and (**F**). P4Qt at pH 7.4 and 5.4 in PBS 1X and in Krebs buffer.

The release profiles of the free quercetin and quercetin encapsulated in the NEs in the PBS1X and Krebs buffers at pH 7.4 and 5.4 are shown in Figure 7. Table S1 compares the % quercetin release at 8 h, and Table S2 compares the quercetin release profiles using the similarity factor (ƒ2). In the case of free quercetin, in both buffers, the % release at 8 h was significantly higher (*p* < 0.05) at pH 7.4 than 5.4 (Table S1), due to the greater solubility of Qt at neutral–alkaline pH values [67]. The pKa of Qt has been reported to be 6.31 [65]. Therefore, the ionized fraction calculated with the Henderson–Hasselbalch equation at pH 7.4 was 92.5%; at pH 5.4, it was only 11%, which explains the results obtained. Concerning the release profile, this effect of the pH was only revealed in PBS1X—see Table S2. The release profile of Qt for the NEs using T80 as a surfactant (F1) is shown in Figure 7B. The % release of Qt at 8 h was significantly lower compared to that of free quercetin, and there was no effect of the pH in either of the buffers—see Table S1. Table S2 shows a significant effect of the buffer on the release profile of Qt, but there was no effect on the pH. It has been described that Qt, in the presence of metal ions (K^+^, Mg^+2^, Ca^+2^), forms complexes that can shield its charge, decreasing its solubility [68]. This effect was evidenced in all formulations by the lower release values of the free and encapsulated Qt in the Krebs buffer (1.18 mM KH_2_PO_4_, 24 mM NaHCO_3_, 118.07 mM NaCl, 4.69 mM KCl, 2.52 mM, CaCl_2_, 1.18 mM MgSO_4_·7H_2_O, pH 7.4), because the Krebs buffer had a higher ionic strength (0.161 mM) than the compendial buffer (0.129 mM) at pH 7.4 [21].

The % release at 8 h for F4Qt and F5Qt, using QPA/Tw80 as a surfactant, was lower than that for F1Qt. Moreover, the % release at pH 5.4 was higher compared to pH 7.4, in both buffers. See Table S1 and Figure 7C,D. Elbialy et al. [68] obtained a similar result when encapsulating curcumin in casein/alginate folate nanoparticles, achieving the more significant release of the active ingredient in a tumor microenvironment stabilized by PBS1X buffer at pH 5.5 (35%), in comparison with pH 7.4. (18%). Additionally, at pH 5.4, QP is close to its pI (4.5), which reduces the protein’s net charge, weakening the intermolecular interactions (electrostatic, hydrogen bonding, and hydrophobic) between the QP and alginate [15,17]. Table S2 shows that F4Qt had the same behavior as F1Qt, with no difference (f2 > 50) due to the pH but a significant difference due to the buffer composition at both pH values. However, in the case of F5Qt, which had a larger proportion of QPA (QPA/T80 = 70/30) compared to F4Qt (QPA/T80 = 60/40), there was only a significant difference (ƒ2 = 48) between the buffers at pH 5.4.

P4Qt and P5Qt, which had QP/Tw80 as the surfactant, did not significantly differ in their release profiles (ƒ2 > 50) regarding the pH or buffer composition—see Table S2. The % release at pH 5.4 was higher compared to pH 7.4 in both buffers, showing similar behavior for F4Qt and F5Qt. See Table S1 and Figure 7E,F.

The in vitro Qt dissolution of F4Qt, F5Qt, P4Qt, and P5Qt in PBS1X and Krebs buffer fits the Korsmeyer–Peppas model, capable of describing the release of drugs from polymers such as the QPA complex and QP, considering non-Fickian mechanisms [69,70]. See Table S3. At pH 7.4 in both buffers, the release exponent (*n*) presented values between 0.5 < *n* < 1. Therefore, the release of Qt was dominated by the swelling and diffusion mechanisms of the polymers. At pH 5.4, the NEs stabilized by the QPA complex (F4Qt and F5Qt) presented *n* > 1, indicating tension and breakage of the polymers (cracking) as a Qt release mechanism, due to the strong effect (pH) of the medium on the polymer [71]. Furthermore, the T50 values estimated from the Korsmeyer–Peppas model showed a decrease when the NEs were brought to a pH of 5.4. F4Qt and P4Qt presented a T50 of 21.7 and 29.3 h in Krebs buffer at pH 7.4 and decreased to 19.3 and 21.3 h when brought to pH 5.4, respectively. A similar effect is also shown in PBS1X (Table S3).

Finally, it is shown that F1Qt presented a higher Qt release rate than F4Qt and P4Qt at pH 7.4. In the ATR-FTIR analysis, F1Qt showed a weak hydrogen bond and hydrophobic interaction between Tw80 and Qt, facilitating the diffusion of Qt to the medium. For this reason, the strong intermolecular interactions between the QPA complex and Qt, evidenced in the R1 (OH and NH stretching) and R2 (changes and displacement of the Amide I and Amide II bands) regions would explain the lower Qt release rates in F4Qt and P4Qt compared to F1Qt, an effect confirmed in the DSC analysis. This is evidenced by the low T50 values of F1Qt (8.5 h) compared to F4Qt (10.4 h) and P4Qt (12.9 h). Similar results were reported by Andrade et al. [70] who used nanoencapsulation of a maqui extract in chitosan/tpp and QP nanovehicles to obtain a higher T50 (5.46 h) for the formulation with a strong intermolecular interaction with the encapsulated active ingredient. Additionally, it is reported that there is a greater release of Qt by diffusion at pH 5.4 compared to pH 7.4 from the NEs F4Qt and P4Qt. This is explained by the loss of QPA and QP complex solubility when found at pH values (2–5) close to the pI of QP and pKa of the alginate, which leads to conformational changes (helix to beta sheets) in the structure of the protein, favoring the release of Qt.

#### 3.8.2. Release in Presence of *B. thetaiotaomicron* and *E. coli*

**Figure 8:**
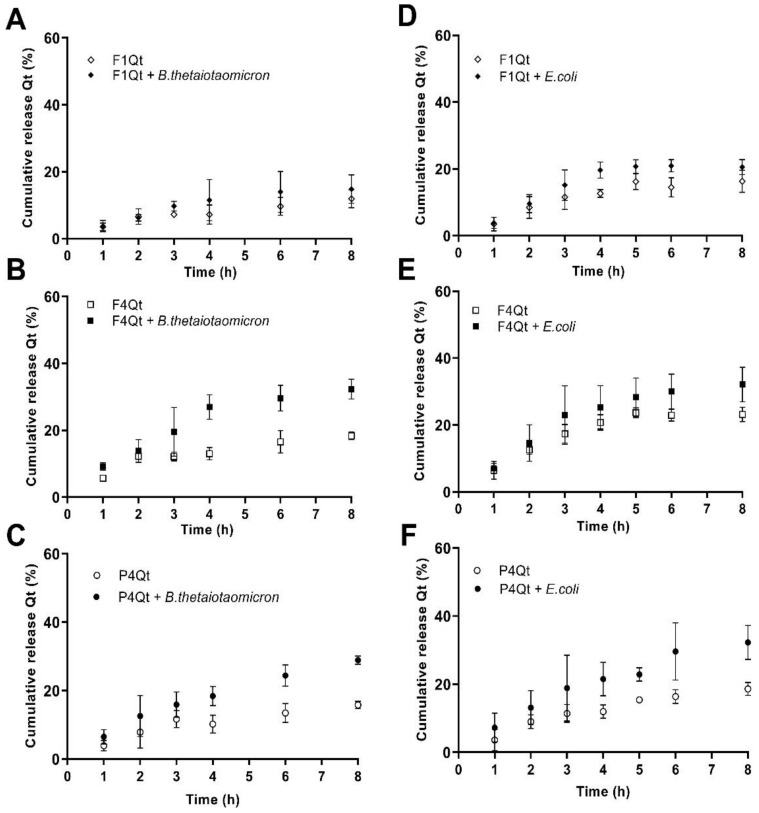
Quercetin release profiles of of (**A**). F1Qt, (**B**). F4Qt, and (**C**). P4Qt in the presence of *B. thetaiotaomicron* (**left**) and of (**D**). F1Qt, (**E**). F4Qt, and (**F**). P4Qt in the presence of *E. coli* (**right**).

The release of Qt from F1Qt, F4Qt, and P4Qt Was evaluated in the presence of *B. thetaiotaomicron* (phylum Bacteroidetes) (see Figure 8A–C) and *E.coli* (phylum Proteobacteria) (see Figure 8D–F), which are generally present in high concentrations in IBD [5]. The release of Qt in the same culture in the absence of bacteria was studied as a control.

In the presence and absence of *B. thetaiotaomicron* (Figure 8A) and *E. coli* (Figure 8D), F1Qt did not present a significant difference in the release of Qt compared to its control (without bacteria) at 8 h of study, reaching a maximum release value of 20.24%. The ƒ2 calculated for F1Qt in the presence and absence of *B. thetaiotaomicron* and *E. coli* was 73 and 69, respectively; therefore, the profiles were considered similar. This occurred because these bacteria did not have the necessary enzymes to metabolize the Tw80 surfactant that stabilized the NE. The lower Qt release values in the control *B. thetaiotaomicron* brain–heart broth may have been due to the higher viscosity of the medium compared to the LB used for *E. coli*.

P4Qt presented a Qt release value of 28.93% in the presence of *B. thetaiotaomicron* at 8 h of study, with a significant difference (*p* < 0.05) versus the control (without bacteria) (Figure 8C). It has been described that, under the conditions of environmental stress (the presence of oxygen), *B. thetaiotaomicron* can easily express four C10 protease genes, degrading the protein of the QP/Tw80 surfactant system and favoring the release of Qt [72]. Likewise, in stress conditions, *E. coli* is able to activate the production of proteases at the extracellular level for better protein assimilation [73,74]. This can explain the higher percentage of Qt release of 32.26% in the presence of *E. coli*, with a significant difference (*p* < 0.05) compared to the control (without bacteria) (Figure 8F). The ƒ2 values calculated for the two graphs were 54 and 51 (ƒ2 < 50), indicating slight similarity between the release profiles in the presence and absence of the microorganisms, respectively. However, the significant difference (*p* < 0.05) in the release of Qt after 4 h of study suggests that the greater release of Qt can be achieved with a longer period of exposure to the microorganism.

F4Qt presented the highest Qt release of 32.34% in the presence of *B. thetaiotaomicron* at 8.0 h of study, with a significant difference (*p* < 0.05) compared to the control (without bacteria) (Figure 8B). The ƒ2 value obtained was 49 (ƒ2 < 50), indicating a difference in the release profiles in the presence and absence of the microorganism. This can be explained by the fact that *B. thetaiotaomicron* is a common intestinal bacterium capable of degrading polysaccharides, such as alginate, which constitutes the QPA complex. This is due to the alginate lyase within it, which decomposes the alginate through a β-elimination reaction, breaking the bonds (1→4) between β-D-mannuronate and α-L-guluronate or β-D-mannuronate and generating oligosaccharides with 4-deoxy-α-L-erythro-hex-4-enuronosyl at their non-reducing ends and β-D-mannuronate at the reducing end [75]. Additionally, the proteolytic activity of the microorganism ensures the degradation of the residual QP that constitutes the QPA complex, favoring the greater release of Qt. In the presence of *E. coli*, the NE stabilized by the QPA/Tw80 surfactant system presented the maximum release of 30.59% at 8.0 h of study, with a significant difference from the control (without bacteria). The ƒ2 value was 64, indicating similarity in the release profiles, showing a sinificant increase only at 8.0 h of study. Other studies have shown that alginate nanovehicles allow the greater release of active ingredients of colonic interest, such as prednisolone and inulin, in the presence of *E coli* [21,76].

The in vitro dissolution of Qt from F4Qt and P4Qt in the presence of *B. thetaiotaomicron* and *E. coli* fit well with the Korsmeyer–Peppas model; see Table S4. According to the morphology of the NEs (spheres) and the “*n*” value (0.43 < *n* < 85), the release mechanism of Qt was by diffusion and swelling of the polymer [69]. Swelling of the QPA complex at pH 7.4 may favor its biodegradability, triggering the release of Qt. F4Qt reduced (∼50%) the T50 value estimated in the presence of *B. thetaiotaomicron* (6.8 h) and *E. coli* (10.9 h), possibly due to the enzymatic action of alginate lyases and proteases present in the microorganisms, respectively. A similar effect was evidenced for NEs stabilized by the QP/tween80 (P4Qt) surfactant system.

In summary, the release of Qt from F1Qt, F4Qt, and P4Qt in the control experiments (without bacteria) is due to the effect of the ionic strength (NaCl and phosphates from the culture medium) on the surfactant systems QPA/Tw80 and QP/tw80, favoring the spread of Qt. A similar effect was observed in the test carried out only with the buffers. On the other hand, F4Qt and P4Qt presented greater release (*p* < 0.05) of Qt than F1Qt due to the swelling of the QPA complex and QP that covers the nanodroplet, strongly hydrophilic polymers, favoring the activity of proteases and alginate lyases of the colonic microbiota healthy as *B. thetaiotaomicron* and in IBD conditions as with *E. coli*. The effect of swelling of the QPA complex and enzymatic activity of the microbiota was evident in the decrease in T50 values (<10 h), triggering the diffusion of Qt by non-Fickian mechanisms (Korsmeyer–Peppas model) [70,71].

### 3.9. Cytotoxicity of NEs in HT-29 Cell Line

**Figure 9:**
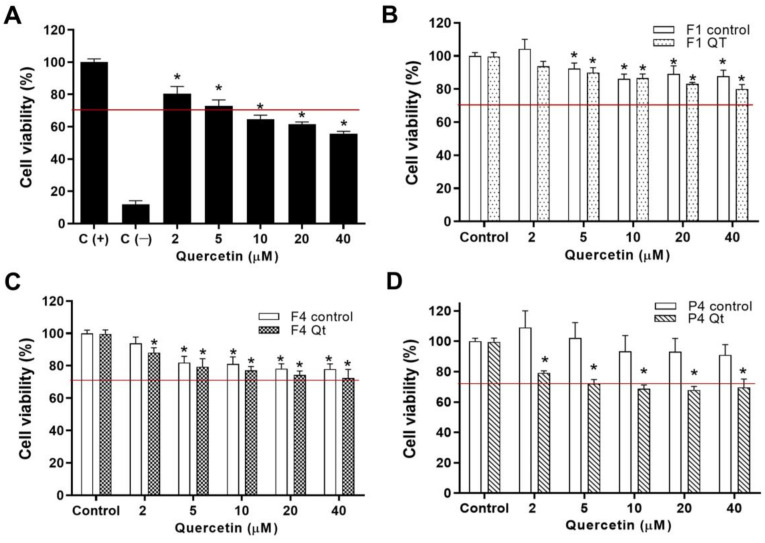
Cell viability of HT-29 at 24 h of culture in the presence of (**A**). Free Qt, (**B**). F1 control and F1Qt, (**C**). F4 control and F4Qt, and (**D**). P4 control and P4Qt, at quercetin concentrations of 2–40 µM. * *p* < 0.05 vs. positive growth control (C+) and red line (cell viability 70%).

The cytotoxicity of free Qt, the NEs without Qt (F1, F4, P4), and the NEs loaded with Qt (F1Qt, F4Qt, P4Qt) against the colon cell line HT-29 was determined, at a final concentration per well of 2 to 40 µM of Qt, for a period of 24 h. The DMEM culture medium that was used to dissolve the NEs was used as a positive control (C+) for cell viability, and 100X 10% triton was used as a negative control (C−)—see Figure 9A.

The HT-29 cells showed dose-dependent sensitivity to the free Qt concentrations, maintaining cell viability between 80.42 and 55.77%, respectively. As shown in Figure 9A, using linear regression, the IC50 was estimated at 45.76 µM, a result similar to those reported by Kaindl et al. [77] and Raja et al. [78], with IC50 values of 50 µM and 42.5 µM required to reduce the viability of HT-29 cells, respectively. It has been shown that Qt can decrease the viability of colorectal cancer cells (CT26, HT-29) by regulating angiogenesis, apoptosis (via caspases and MAPK), and cell cycle arrest (G1-S) [79].

F1, F4, and P4 were not cytotoxic against the HT-29 cell line and can be considered safe according to ISO standard [80] as they maintain cell viability greater than 70% (red line)—see Figure 9B–D. F1 did not show sufficient cytotoxicity to reduce the cell viability, despite using a synthetic surfactant, possibly due to the low concentration of Tw80 (2% *w*/*v*) used in the initial formulation. F4 and P4 did not show cytotoxicity, as expected, because they used mainly natural surfactants.

At a concentration of 5 µM of Qt in F1Qt, the viability of HT-29 cells was significantly reduced (*p* < 0.05) compared to the growth control (C+); meanwhile, at 20 and 40 µM of Qt, there was a significant difference (*p* < 0.05) compared to the F1 control. F4Qt and P4Qt significantly reduced the cell viability to 69.53 and 64.47% compared to their control NEs at the highest concentration of Qt, respectively. The decrease in the cell viability of HT-29 in the presence of F1Qt, F4Qt, and P4Qt was due to the controlled release of Qt, which has a toxic effect on this type of cell. The IC50 values of F1Qt, F4Qt, and P4Qt were 97.30, 89.82, and 83.63 µM, respectively. These values were significantly (*p* < 0.05) higher than those of non-encapsulated Qt. This is because non-encapsulated Qt is fully available to interact with HT-29 cells, rapidly reducing their viability, while the NEs presented the slow and controlled release of the active ingredient. F4Qt and P4Qt had a greater cytotoxic effect on the cells compared to F1Qt, which could have been due to their greater antioxidant and anti-inflammatory capacity compared to free Qt (see Section 3.10). It has been determined that compounds with antioxidant and anti-inflammatory properties reduce the viability of cells of cancerous origin, such as HT-29 [81].

### 3.10. Antioxidant Capacity of Qt and NEs Measured via ABTS and ORAC

According to the ABTS and ORAC methods, the antioxidant capacity of unencapsulated Qt was 7.57 µmol TE/mg quercetin and 50.01 µmol TE/mg quercetin—see Table 3. In the ABTS assay, F4Qt showed an ABTS value (7.52 µmol ET/mg Qt) higher (*p* < 0.05) than that of F1Qt and similar to that of free Qt; the same profile was evidenced for the IC50 value (2.14 µg/mL), i.e., the concentration necessary to inhibit 50% of the ABTS·+ radical. The increase in antioxidant capacity may have been due to the presence of QP in the QPA/Tw80 surfactant system. It has been reported that proteins and peptides of plant origin have the ability to block the ABTS·+ radical thanks to the transfer of electrons or hydrogen by different functional groups (hydroxyl, imino, aromatic) present in the amino acid side chain [25,82]. Amino acids such as Trp, Tyr, Met, Cys, His, and Phe have an antioxidant capacity and are present in quinoa QP [46,81]. The unfolding and partial denaturation of proteins at the O/W interface exposes the internal hydrophobic regions of the protein, mainly aromatic amino acids, resulting in increased bioactivity [83,84]. The high values of superficial hydrophobicity reported by Arazo et al. [17] and in the FTIR analyses in this study support this explanation. Martínez et al. [84] used quinoa chenopodin as a nanovehicle to encapsulate betanin, increasing its ABTS antioxidant capacity by 1.5 times compared to free betanin. The additive effect of QP on the antioxidant capacity was also evident in P4Qt (7.92 µmol ET/mg Qt), where it was significantly higher (*p* < 0.05) compared to that of free Qt, F1Qt, and F4Qt, possibly because P4Qt had 1.16 times more QP in the surfactant system than F4Qt.

The ORAC values presented a similar profile to the results of the ABTS assay. F4Qt showed greater (*p* < 0.05) antioxidant capacity than free Qt and F1Qt, with a value of 55.33 µmol ET/mg of Qt. The additive effect of QP on the antioxidant capacity of NEs stabilized by QPA/Tw80 and QP/Tw80 became more significant because ORAC is a highly sensitive fluorescent assay that uses peroxyl radicals as an oxidizing agent [85]. Peptides and proteins exhibit antioxidant capacity in this assay due to the amino acids Trp, Tyr, Met, Cys, and His present in QP, whose primary mechanism is the donation of protons to peroxyl radicals [25,84].

**Figure 10:**
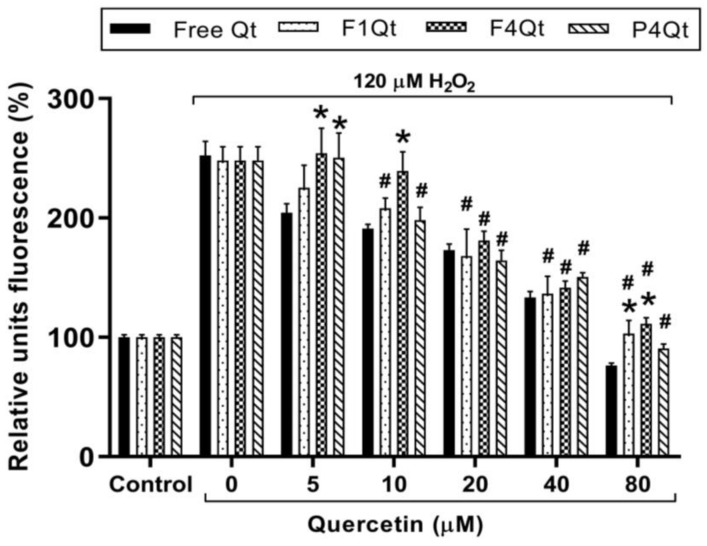
Effects of free Qt, F1Qt, F4Qt, and P4Qt at different concentrations of Qt (5–80 µM) against HT-29 cells treated with 120 µM H_2_O_2_. * indicates *p* < 0.05 vs. free Qt; # indicates *p* < 0.05 vs. 120 µM H_2_O_2_ without antioxidant.

The ability of F1Qt, F4Qt, and P4Qt to neutralize reactive oxygen species (ROS) at the intracellular level in HT-29 cells was determined via the dichlorofluorescein diacetate oxidation method (DCFH-DA) [26]. DCFH-DA is a non-fluorescent compound that penetrates cells and is converted into DCFH via the action of plasma esterases; when in contact with ROS, it becomes DCF (fluorescent). In Figure 10, the relative fluorescence values generated via the formation of DCF are shown. The fluorescence generated by the basal presence of ROS in the cells was established as the base URF (100%), which, when adding H_2_O_2_ (120 µM), increased to 253%. When adding free Qt, the fluorescence was reduced depending on the applied dose (5 to 80 µM), with the URF values decreasing from 204 to 76%, respectively. F4Qt and P4Qt reduced the URF values of the loaded Qt (5–80 µM) in a dose-dependent manner in the range of 254 to 111% and 250 to 91%, respectively. F4Qt presented a similar ability to block ROS to free Qt at Qt concentrations of 20 and 40 µM, without significant differences (*p* < 0.05). In a linear projection of the antioxidant capacity, F4Qt needed a concentration of 92.65 µM to thoroughly neutralize the oxidative stress generated by the addition of H_2_O_2_, with no significant difference compared to that reported for F1Qt of 89.62 µM but higher (*p* < 0.05) than that measured for P4Qt at 76.06 µM. These results coincide with those of the ORAC assay, where P4Qt presented greater antioxidant capacity thanks to the greater presence of QP. The NEs required higher concentrations of Qt to thoroughly neutralize the oxidative stress generated by H_2_O_2_, compared to non-encapsulated Qt. This was probably because the non-encapsulated Qt was able to penetrate the HT-29 cells during the 6.0 h period of exposure; meanwhile, Qt could not be fully released from the NEs. It should be noted that the effective Qt concentrations required to neutralize the high oxidative stress presented by F4Qt remained in the range (10–100 µM) of effective Qt required to reverse oxidative and inflammatory stress conditions in studies related to IBD [77,78]. P4Qt is also presented as an alternative to nanovehicles for controlled colonic delivery.

## 4. Conclusions

NEs based mainly on a soluble QPA (chenopodin/alginate) complex or chenopodin showed high efficiency in the encapsulation of quercetin; they were stable under simulated gastrointestinal tract conditions, but not at acidic pH levels close to the pI (4.5) of QP and below the pKa (3.0–3.5) of the carboxylate group of alginate. Quercetin release from the NEs was triggered by *B. thetaiotaomicron*, and the nanovehicles did not show cytotoxicity in the HT-29 cell line. The antioxidant capacity of the Nes, as measured via the ABTS and ORAC methods, increased due to the presence of QP in the vehicle. The capacity to neutralize reactive oxygen species (ROS) at the intracellular level in HT-29 cells also increased, as determined by the dichlorofluorescein diacetate oxidation method (DCFH-DA).

## Figures and Tables

**Table 1:** Comparison of the crossflow ultrafiltration processes of methods 1 and 2, using 30 and 100 kDa membranes.

	Crossflow UF Method 1 *		Crossflow UF Method 2 ^this work^	
	DF	Concentration	DF	Concentration
	30 and 100 kDa	100 kDa	100–30 kDa	100–30 kDa
Feed volume (mL)	200	200	200	400
Operating time (h)	1.32	0.06	0.76	0.16
Final retained volume (mL)	200	40	200	50
Soluble proteins in final concentration stage (mg/mL)		21.11		42.7
Total protein in final concentrate (g)		0.844		2.14
Protein yield (% *w*/*w*)		26.14		32.75

* Reported by Arazo et al. [16].

**Table 2:** Size, PDI, and zeta potential of NE controls and NE-Qt samples.

NE Control	Ratio QPA/Tw80	Size (nm)	PDI	Zeta Potential	EE (%)	LC (mg/mL)
F1	0/100	170.7 ± 1.5 a	0.215 ± 0.006 a	−5.67 ± 0.58 a	N.D.	N.D.
F2	20/80	178.3 ± 2.5 b	0.241 ± 0.013 b	−7.97 ± 0.26 b	N.D.	N.D.
F3	40/60	201.3 ± 3.5 c	0.238 ± 0.010 b	−8.31 ± 0.55 b	N.D.	N.D.
F4	60/40	219.3 ± 1.5 d	0.237 ± 0.009 b	−9.40 ± 1.58 b	N.D.	N.D.
F5	70/30	248.0 ± 10.8 e	0.245 ± 0.022 b	−11.20 ± 1.00 bc	N.D.	N.D.
F6	80/20	263.0 ± 2.6 f	0.252 ± 0.013 b	−14.50 ± 1.36 cd	N.D.	N.D.
NE-Qt						
F1Qt	0/100	160.1 ± 2.2 a	0.232 ± 0.013 a	−13.03 ± 1.81 a	90.78 ± 3.77 a	0.91 ± 0.04 a
F2Qt	20/80	175.3 ± 1.2 b	0.282 ± 0.012 b	−18.33 ± 1.58 b	89.83 ± 5.91 a	0.90 ± 0.06 a
F3Qt	40/60	202.3 ± 4.2 c	0.270 ± 0.010 b	−19.00 ± 1.73 b	88.90 ± 6.11 a	0.89 ± 0.06 a
F4Qt	60/40	226.8 ± 3.2 d	0.249 ± 0.021 a	−20.57 ± 1.75 b	87.66 ± 2.69 a	0.88 ± 0.03 a
F5Qt	70/30	257.8 ± 2.5 e	0.261 ± 0.014 b	−22.13 ± 1.25 bc	85.72 ± 3.82 a	0.86 ± 0.04 a
F6Qt	80/20	314.8 ± 7.8 f	0.258 ± 0.012 b	−24.90 ± 1.65 cd	86.00 ± 3.43 a	0.86 ± 0.02 a

Different letters (a–f) in the same column represent significant differences (*p* < 0.05).

**Table 3:** Antioxidant activity of free Qt and NEs F1-Qt, F4-Qt, and P4-Qt, evaluated via ABTS and ORAC.

	ABTS		ORAC
	µmol TE/mg of Quercetin	IC50 (µg/mL)	µmol TE/mg of Quercetin
Free Qt	7.57 ± 0.19 a	2.13 ± 0.01 a	50.01 ± 2.23 a
F1Qt	7.01 ± 0.09 b	2.44 ± 0.05 b	51.76 ± 1.25 a
F4Qt	7.52 ± 0.10 a	2.14 ± 0.08 a	55.33 ± 2.70 b
P4Qt	7.92 ± 0.40 ac	2.11 ± 0.03 a	56.85 ± 5.32 ab

Different letters (a–c) in the same column represent significant differences (*p* < 0.05).

## Data Availability

Data are contained within the article.

## Supplementary Materials

The following supporting information can be downloaded at: https://www.mdpi.com/article/10.3390/antiox13060658/s1, Figure S1: Determination of calculated values of size, PDI and zeta potential of the QPA complex. Table S1: Release of Qt at 8 h of study from the formulations; Table S2: Release profile similarity (f2); Table S3: Parameters of the Korsmeyer-Peppas mathematical model for Qt release profiles in PBS1X and Krebs buffer at pH 7.4 and 5.4; Table S4: Parameters of the Korsmeyer-Peppas mathematical model for Qt release profiles in the presence of *B. thetaiotaomicron* and *E. coli*.
