# Supplementary material for: Nanoemulsions Based on Soluble Chenopodin/Alginate Complex for Colonic Delivery of Quercetin
Source: Antioxidants (Basel). 2024 May 27;13(6):658. doi: 10.3390/antiox13060658 (PMC11200757; doi:10.3390/antiox13060658)
Supplement: Supplementary file 1 [file antioxidants-13-00658-s001.zip › antioxidants-2997673-supplementary.pdf]

Supplementary material

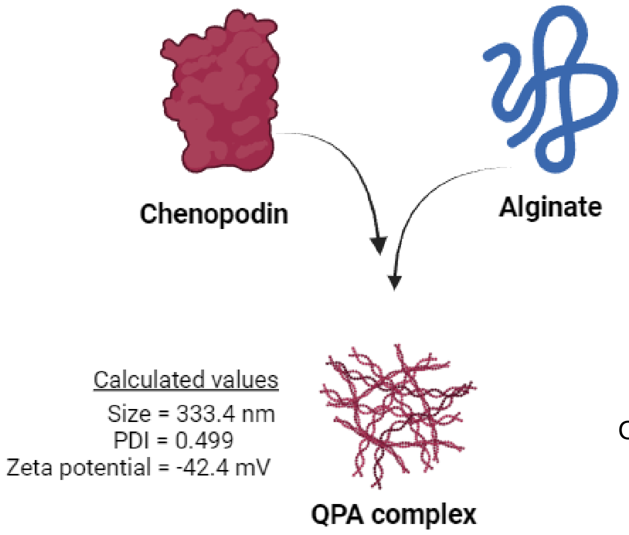

	Size (nm)	PDI	Zeta potential (mV)	Proportion in QPA complex
Chenopodin (QP)	59.8	0.421	-31.0	0.6
Alginate (Alg)	743.9	0.616	-59.6	0.4

***** Evaluated at pH 6.0 and 25°C

Calculation example:

$$\text{Calculated size*} = \left(\text{QP size} \times \text{Proportion of QP in the QPA complex}\right) + \left(\text{alginate size} \times \text{Proportion of Alg in the QPA complex}\right)$$

***** Calculated PDI and calculated zeta potential are obtained in the same way

Figure S1. Determination of calculated values of size, PDI and zeta potential of the QPA complex

Table S1. Release of Qt at 8 h of study from the formulations

	% Release at 8 h (Mean ± SD)			
	PBS1X		Krebs	
**Sample**	**pH 7.4**	**pH 5.4**	**pH 7.4**	**pH 5.4**
**Qt libre**	70.48 ± 4.90[a]	62.45 ± 0.45[a*]	64.13 ± 4.95[a]	57.29 ± 3.13[a*]
**F1Qt**	36.13 ± 1.91[b]	35.86 ± 2.49[b]	21.69 ± 1.99[b]	20.32 ± 0.25[b]
**F4Qt**	28.74 ± 5.87[c]	35.90 ± 3.89[b*]	10.76 ± 0.48[c]	14.25 ± 0.74[b]
**F5Qt**	24.56 ± 8.04[c]	32.02 ± 5.10[b*]	10.05 ± 0.40[c]	13.89 ± 0.54[b]
**P4Qt**	22.88 ± 1.60[c]	32.55 ± 0.46[b*]	14.13 ± 0.39[c]	17.94 ± 0.37[b]
**P5Qt**	20.73 ± 0.72[c]	27.61 ± 1.91[c*]	11.83 ± 0.94[c]	15.91 ± 0.81[b]

[a, b, c.] Different letters in the same column represent a significant difference ($p<0.05$)

* significant difference vs pH 7.4 in the same buffer ($p<0.05$)

Table S2. Release profile similarity (f2)

Formulations	*ƒ*2 value	Formulations	*ƒ*2 value
**Free Qt**		**P4Qt with QP/Tw80 (60/40)**	
PBS1X-7.4 and PBS1X-5.4	48	PBS1X-7.4 and PBS1X-5.4	64
Krebs-7.4 and Krebs-5.4	53	Krebs-7.4 and Krebs-5.4	78
PBS1X-7.4 and Krebs-7.4	53	PBS1X-7.4 and Krebs-7.4	55
PBS1X-5.4 and Krebs-5.4	57	PBS1X-5.4 and Krebs-5.4	50
**F1Qt with Tw80**		**F5Qt with QPA/Tw80 (70/30)**	
PBS1X-7.4 and PBS1X-5.4	73	PBS1X-7.4 and PBS1X-5.4	69
Krebs-7.4 and Krebs-5.4	88	Krebs-7.4 and Krebs-5.4	83
PBS1X-7.4 and Krebs-7.4	43	PBS1X-7.4 and Krebs-7.4	50
PBS1X-5.4 and Krebs-5.4	41	PBS1X-5.4 and Krebs-5.4	48
**F4Qt with QPA/Tw80 (60/40)**		**P5Qt with QP/T80 (70/30)**	
PBS1X-7.4 and PBS1X-5.4	71	PBS1X-7.4 and PBS1X-5.4	72
Krebs-7.4 and Krebs-5.4	84	Krebs-7.4 and Krebs-5.4	81
PBS1X-7.4 and Krebs-7.4	46	PBS1X-7.4 and Krebs-7.4	56
PBS1X-5.4 and Krebs-5.4	45	PBS1X-5.4 and Krebs-5.4	54

Table S3. Parameters of the Korsmeyer-Peppas mathematical model for Qt release profiles in PBS1X and Krebs buffer at pH 7.4 and 5.4.

	**PBS1X**						**Krebs**					
	**pH 7.4**			**pH 5.4**			**pH 7.4**			**pH 5.4**		
	**n**	**R^2**	**T50 (h)**	**n**	**R^2**	**T50 (h)**	**n**	**R^2**	**T50 (h)**	**n**	**R^2**	**T50 (h)**
F1Qt	0.8499	0.9163	8.5	1.0102	0.8548	8.69	0.9377	0.9781	18.4	0.9871	0.9428	18.2
F4Qt	1.074	0.9194	10.4	1.0968	0.9836	9.1	1.2021	0.9338	21.7	1.2394	0.9767	19.3
F5Qt	0.8624	0.9285	15.2	1.2558	0.9640	8.5	0.8810	0.953	42.4	1.1893	0.9777	21.5
P4Qt	0.8302	0.9960	12.9	0.8801	0.9506	9.4	0.8188	0.931	29.3	0.9188	0.9483	21.3
P5Qt	0.7955	0.9567	14.5	0.8079	0.9771	13.0	0.8488	0.9213	28.5	0.8770	0.982	25.7

"n" is the exponent of release of the mathematical model.

Table S4. Parameters of the Korsmeyer-Peppas mathematical model for Qt release profiles in the presence of *B. thetaiotaomicron* and *E. coli*.

	B. thetaiotaomicron			***E. coli.***		
	**n**	**R^2**	**T50 (h)**	**n**	**R^2**	**T50 (h)**
F1Qt control	0.7065	0.9683	28.5	0.8822	0.9047	19.1
F4Qt control	0.4897	0.9739	20.3	0.6285	0.9597	18.0
P4Qt control	0.6735	0.9628	19.6	0.8219	0.9586	21.3
F1Qt + bacteria	0.6308	0.9454	20.6	0.6974	0.9722	18.9
F4Qt + bacteria	0.6416	0.9891	6.2	0.7302	0.9827	10.9
P4Qt + bacteria	0.699	0.9924	8	0.8125	0.9849	11.3

"n" is the exponent of release of the mathematical model.
